# In Silico Regression Modeling and Improved Interpretability To Predict the Transport Inhibitory Activity of Breast Cancer Resistance Protein

**DOI:** 10.1021/acsomega.5c12191

**Published:** 2026-04-21

**Authors:** Kaoru Takadera, Donny Ramadhan, Reiko Watanabe, Kenji Mizuguchi

**Affiliations:** † Laboratory for Computational Biology, Institute for Protein Research, The University of Osaka, Suita, Osaka 565-0871, Japan; ‡ Graduate School of Pharmaceutical Sciences, The University of Osaka, Suita, Osaka 565-0871, Japan; § Artificial Intelligence Center for Health and Biomedical Research, National Institutes of Biomedical Innovation, Health and Nutrition, Settsu, Osaka 566-0002, Japan; ∥ Graduate School of Science, The University of Osaka, Toyonaka, Osaka 560-0043, Japan; ⊥ Research Center for Pharmaceutical Ingredients and Traditional Medicine, National Research and Innovation Agency (BRIN), Bogor, West Java 16911, Indonesia

## Abstract

Breast cancer-resistance protein (BCRP) functions as an efflux transporter, and its inhibition is an important pharmacokinetic parameter in drug–drug interactions (DDIs). In silico approaches that predict compound profiles from chemical structures are widely used in early drug discovery, and binary classification models have been developed for BCRP inhibition. While classification models distinguish inhibitors from noninhibitors, regression models provide numerical predictions that enable ranking. Additionally, there has been a growing demand for models with better interpretability. In this study, we aimed to construct regression models that can predict the IC_50_ values of BCRP inhibitory activity and to interpret the predicted results. Prediction models were built using a high-quality data set comprising 870 compounds with IC_50_ values obtained from publicly available sources, and the best model achieved an *R* ^2^ value of 0.736 on the test set. Although the model demonstrated satisfactory performance, the use of descriptors that were difficult to interpret was found to limit the explainability of its predictions. To overcome this problem, we applied a new set of machine learning models using only substructure-based fingerprints and a tree-based algorithm. The best model showed an *R* ^2^ value of 0.703 on the test set. Based on SHapley Additive exPlanations (SHAP) values, several substructures were identified as key features by the finalized model, and the prediction results revealed seven potential BCRP inhibitors among approved drugs. These models contribute to accelerating compound screening and reducing drug development costs, while also aiding in the exclusion of potential dropout candidates.

## Graphical abstract

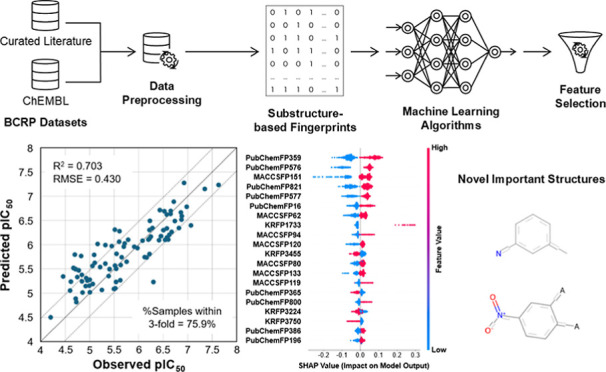

## Introduction

Recently, the application of machine learning (ML) and simulation technologies in drug discovery has reduced the need for animal experiments and has substantially contributed to the automation and acceleration of the drug discovery process while lowering costs.^1^ The prediction of compound profiles using ML is crucial in the early stages of drug discovery, and new methods for in silico screening of large libraries have been developed for practical use owing to advances in information science, technology, and computational power. Absorption, distribution, metabolism, excretion, and toxicity (ADMET) are key factors in drug design. Failure to meet the required ADMET criteria is a common cause of the high attrition rates among drug candidates.^2^ Therefore, various in vitro ADMET screening assays have been developed, which have contributed to the available experimental data; however, these are expensive, especially when thousands of compounds are involved.^3^ ML has attracted substantial attention and is expected to become an indispensable technology for efficiently designing drugs with appropriate ADMET properties and facilitating lead compound optimization.^4^ Lead optimization is a critical phase of drug discovery in which biologically active hits are chemically modified to enhance their ADMET properties, thereby generating more promising drug candidates.^5^

The blood–brain barrier (BBB) serves as a biological barrier between the blood circulation and the central nervous system (CNS). It plays a critical role as a gatekeeper of brain function by regulating the efflux of nutrients, metabolites, and drugs. Impairment of BBB function contributes to the pathology of neurological conditions, including multiple sclerosis, stroke, epilepsy, and Alzheimer’s disease.^6^ In the BBB, efflux transporters expressed on the apical membrane actively pump out unnecessary substances, such as therapeutic drugs, endogenous metabolites, and environmental toxicants, back into the bloodstream, thereby protecting the brain.^7^ One group of BBB transporters comprises the ATP-binding cassette (ABC) transporters, including the breast cancer resistance protein (BCRP), which is encoded by the ATP-binding cassette subfamily G member 2 (ABCG2) gene. BCRP is an efflux transporter that actively exports various endogenous and exogenous substrates, including drugs, from the brain to the blood, thereby maintaining brain homeostasis and protecting the brain against xenobiotics. Although BCRP is not exclusive to the BBB, it is highly expressed in barrier cells, including those found in the colon, small intestine, placenta, and liver. Inhibition of BCRP increases the concentration of drugs in the CNS and is considered an important pharmacokinetic factor in drug–drug interactions (DDIs).^8,9^ BCRP is listed as a transporter that should be carefully evaluated for clinically relevant DDIs by the Pharmaceutical and Life Sanitation Bureau of the Ministry of Health, Labour and Welfare, Japan, the U.S. Food and Drug Administration (FDA),^10^ and the European Medicines Agency (EMA).^11^

Several in silico approaches have been developed to predict BCRP inhibition based on chemical structure information using ML, with most studies reporting classification models. Jiang et al.^12^ developed a series of structure–activity relationship models to distinguish between BCRP inhibitors and noninhibitors. Huang et al.^13^ constructed several ligand-based classification models using partial least-squares-discriminant analysis combined with molecular interaction fields and fingerprint-based structural descriptions to capture physicochemical and fragmental features related to BCRP inhibition, exploiting a large public data set. Several other model-building studies were also reported by Kong et al.^14^ However, previously reported binary classification models do not always use clear thresholds for distinguishing inhibitors from noninhibitors. Some models take class labels from multiple original data sources where the threshold varies, while others combine metrics such as *K*_*i*_ and percentage inhibition without proper conversion. This lack of consistency limits the ability to apply flexible activity thresholds, which are typically selected based on research themes, drug targets, and stages.

One advantage of regression models is their ability to set flexible thresholds by directly predicting numerical values, allowing applications for classification and ranking depending on the research objective. Ding et al.^15^ provided a pharmacophore ensemble/support vector machine (SVM) scheme trained with 22 compounds to predict BCRP inhibition as pIC_50_ values. While the pharmacophore approach has the advantage of facilitating the interpretation of specific interactions, it requires generating 3D structures and manual engineering of protein–ligand interactions for each compound, which limits scalability. In addition, 3D quantitative structure–activity relationship (QSAR) and protein–ligand docking approaches have been used for IC_50_ prediction;^16,17^ however, their practical application is often constrained by relatively high computational cost for large compound libraries and sensitivity to ligand conformational changes.^18^ In this context, constructing a machine-learning regression model that directly predicts numerical IC_50_ values using only 2D structural information represents a practical and efficient alternative for drug development. Furthermore, there has been a growing demand for explainable artificial intelligence (AI) that enables users to understand the rationale behind AI’s decisions.^19^

In this study, we constructed a regression model to predict the pIC_50_ values of BCRP inhibition using an ML approach with only 2D structural information. Simplified predictive models that can be explained by selected features were constructed, and their interpretation revealed substructures that are important for BCRP inhibition. In addition, seven approved drugs were identified as potential inhibitors of BCRP among commercially available drugs.

## Experimental Section

### Data Collection

We collected data to construct three data sets: one for predicting BCRP inhibition, one containing approved drugs, and another consisting of compounds with previously reported BCRP inhibitory activity. The experimental BCRP inhibition data in humans used for model construction and prediction were obtained from the ChEMBL database (version 30)^20^ and Jiang et al.^12^ A total of 1572 IC_50_ values with experimental information were extracted from CHEMBL5393, which was selected as it corresponds to a single, well-defined human ABCG2 protein and provides a comprehensive, well-annotated set of IC_50_ measurements. The structural information on these compounds in the experimental data set was represented using simplified molecular input line entry system (SMILES) format. Additionally, 2799 BCRP inhibition data points were obtained from Jiang et al.

The list and structural information on approved drugs were collected from the KEGG DRUG database^21^ and the ChEMBL database (version 30). PubChem SIDs were extracted from the KEGG DRUG database for organic compounds with commercial drug names, and their corresponding SMILES representations were linked using PubChemPy.^22^ The ChEMBL IDs of the marketed drugs labeled as “max_phase = 4” were also obtained from the ChEMBL database, and their SMILES data were collected. In addition, a list of previously reported BCRP inhibitors containing 148 drugs was obtained from DrugBank^23^ under accession number DBCAT002662 (DBCAT004102).

### Data Curation and Descriptor Calculation

For the BCRP inhibition data set, the collected data were carefully curated to include only those obtained under typical experimental conditions, specifically cell-based transport assays used to evaluate BCRP-mediated substrate transport and its inhibition by chemicals. For the ChEMBL data, the curation process involved tagging experimental details such as assay type, substrates, and cell type, from the data description; filtering and removing data derived from irrelevant experimental conditions; standardizing all measurement units to “μM”; and averaging multiple IC_50_ values for the same compound and manually confirming their consistency (within a 3-fold range) by cross-checking the original sources. As a result, IC_50_ values for 670 compounds with structural information were obtained from ChEMBL.

The data set by Jiang et al. was previously carefully curated. When multiple measurements were available for a single compound, the IC_50_/EC_50_ value was either selected or averaged with reference to the original publication. The curation process involved extracting IC_50_ and EC_50_ values, where EC_50_ values were treated as IC_50_ values after confirming from the original paper that they represented inhibitor potency; removing missing entries and retaining only numerical data; and standardizing all IC_50_/EC_50_ units to “μM”. IC_50_ values for 565 compounds with structural information were obtained from Jiang et al.

The ChEMBL and Jiang et al. data sets were integrated, and the SMILES were standardized using MELLODY-TUNER.^24^ Among them, 363 compounds overlapped, and the average IC_50_ value was used in the final data set after confirming the consistency of IC_50_ values between the ChEMBL and Jiang et al. data by checking the original references. All IC_50_ values were then converted to pIC_50_ (−log_10_ IC_50_ [M]). Ultimately, the curated BCRP inhibition data set comprised 870 compounds and was defined as the BCRP data set. Details of the manual curation procedure are provided in Scheme S1 of Supporting Information.

For approved drugs, the ChEMBL and KEGG data sets were merged after SMILES standardization using MELLODY-TUNER. The resulting data set contained 4301 compounds with compound names and SMILES information and was defined as the approved drug data set.

For previously reported BCRP inhibitors, 114 out of 148 compounds that overlapped with approved drugs were selected. Among them, 13 compounds that were already included in the BCRP data set were removed. Consequently, a list of 101 compounds was created and defined as the list of reported BCRP inhibitors.

The descriptors used for model construction were calculated using RDKit,^25^ Mordred,^26^ PaDEL-Descriptor,^27^ and jCompoundMapper.^28^ A summary of the descriptors is presented in Table S1.

### Data Set Visualization

Principal component analysis (PCA) with the MACCS keys (166 bits) was performed using the scikit-learn package,^29^ retaining components with a cumulative contribution ratio of at least 90%. Subsequently, uniform manifold approximation and projection (UMAP)^30^ was applied using UMAP-learn with default settings to reduce the features to two dimensions for visualization.

### Model Construction

To predict IC_50_ values, ML models were constructed using compound descriptors as input features and IC_50_ values as the output through supervised learning. An overview of the model-building process is shown in Figure S1. The BCRP data set was divided into training and test sets using a repeated random splitting strategy with a 9:1 ratio. This procedure was repeated for 10 iterations using different random seeds, such that each iteration employed an independent train-test split.

A total of 1630 physicochemical descriptors calculated using RDKit and Mordred, and 28,675 molecular fingerprints calculated using jCompoundMapper, were used as input features. Descriptors with low variation among compounds (variance <0.01) were removed, and those with highly similar distributions (correlation coefficient >0.8) were also eliminated. To reduce bias caused by differences in the scale of physicochemical descriptors, the descriptors were normalized to have a mean of 0 and a standard deviation of 1 based on the training set. The Boruta_py package^31^ was used to identify important features by comparing their importance, calculated using random forest (RF),^32^ with that of randomly generated shadow features, selecting those with importance exceeding the 80% threshold.

Hyperparameter optimization was performed using the Optuna package^33^ with Bayesian optimization and 5-fold nested cross-validation. The Optuna settings are summarized in Table S2. The algorithms used for model construction included RF, light gradient boosting machine (LGBM),^34^ SVM,^35^ artificial neural network (ANN),^36^ k-nearest neighbors (kNN), and Ridge and Lasso regression (RL). The constructed models were evaluated on the test set, which was excluded from the training process, using evaluation metrics.

The second model is a simplified descriptor model constructed using structure-based fingerprints such as MACCS, PubChem, and Klekota-Roth fingerprints (KRFP), which were used as input features. Although the overall model construction process was the same as that of the first model, feature reduction was performed to improve model interpretability. Recursive feature elimination (RFE) was applied step by step while monitoring the evolution of prediction accuracy. Using the scikit-learn RandomForestRegressor with max_depth = 6 and n_estimators = 200, the features were first reduced to 700 dimensions and then gradually decreased in steps of 10, from 700 to 20 dimensions. The changes in evaluation results were monitored throughout this process.

We selected the point at which the root mean squared error (RMSE) value began to increase, and the *R*-squared (*R* ^2^) value began to decrease, using the KneeFinder package^37^ to identify the knee (elbow) point. The Optuna settings used for hyperparameter optimization are summarized in Table S3. Three algorithms, RF, LGBM, and LR, were employed for model construction. These tree-based and linear algorithms were selected to enhance the interpretability of the predictive models. The constructed models were evaluated on the test set using evaluation metrics. Finally, after evaluation with the test sets, the second simplified descriptor model was retrained on the entire data set using the best hyperparameter settings identified among the 10 splits and defined as the finalized model.

### Evaluation Metrics

The predictive performance of the model was evaluated using RMSE and *R* ^2^, which were calculated for the test set, as shown in eqs 1 and 2.

1 $$RMSE=\sqrt{\frac{1}{n}\sum_{i=1}^{n}{\left(y_{i}-{ŷ}_{i}\right)}^{2}}$$

2 $$R^{2}=1-\frac{\sum_{i=1}^{n}\left(y_{i}-{ŷ}_{i}\right)}{\sum_{i=1}^{n}\left(y_{i}-\mathrm{y̅}\right)}$$

where *n* is the number of samples, *y*_*i*_ is the actual value, ${ŷ}_{i}$ is the predicted value, and *y̅* is the average value.

The balanced accuracy (BA) and Matthew’s correlation coefficient (MCC) were also used to evaluate the classification tasks, calculated using eqs 3 and 4, respectively.

3 $$BA=0.5\times \left(\frac{TP}{TP+FN}+\frac{TN}{TN+FP}\right)$$

4 $$MCC=\frac{TP\times TN-FN\times FP}{\sqrt{\left(TP+FN\right)\left(TP+FP\right)\left(FN+TN\right)\left(TN+FP\right)}}$$

where TP stands for true positives, which are samples correctly predicted as positive, while TN represents true negatives, referring to samples correctly predicted as negative. FP denotes false positives, which are negative samples incorrectly predicted as positive, and FN refers to false negatives, which are positive samples incorrectly predicted as negative. MCC evaluates classification performance by considering the balance among all four prediction categories, whereas BA represents the average of sensitivity and specificity.

### SHapley Additive exPlanation (SHAP) Analysis

SHAP analysis was used to identify important features by calculating their contributions to the predicted values using the SHAP package.^38^ This analysis quantifies how each input feature influences the model’s predictions. The SHAP values were computed for the finalized model and averaged across the entire BCRP data set to compare the importance of each feature relative to the overall compound.

### Substructures Identification

The substructures corresponding to each fingerprint were identified as SMILES arbitrary target specification (SMARTS) by referring to the PubChem web page^39^ for the PubChem Fingerprint, the Daylight web page^40^ for the Klekota-Roth Fingerprint, and the RDKit MACCS implementation.^41^

### Determination of Tanimoto Coefficient Cutoff for Predicting pIC_50_ Using Approved Drugs

In the 10 different test set splits used for evaluating the finalized model, compounds were classified as TP and FP based on a pIC_50_ threshold of 5. The Tanimoto coefficient was calculated based on Morgan fingerprints generated using RDKit between each compound in the test and training sets. The highest Tanimoto coefficient was determined and defined as the maximum Tanimoto similarity (max Tanimoto). The max Tanimoto values of compounds in the TP and FP groups were plotted, and the cutoff value of max Tanimoto was determined at the point where the false positive rate (FPR) fell below 0.3. Classification to distinguish the TP and FP groups was performed by shifting the max Tanimoto cutoff from 0.4 to 0.8 in 0.01 increments. The cutoff value determined for each test set and the corresponding plots are shown in Figure S2. Finally, the average of the cutoff values across all test sets was defined as the final cutoff.

## Results

### Construction of the Data Set

To create a data set for constructing a predictive model, data on inhibitory activity against BCRP were collected. A total of 1572 experimental data entries for BCRP inhibition were extracted from the ChEMBL database (version 30),^20^ and 2799 in vitro BCRP inhibitory activity values from Jiang et al.^12^ EC_50_ values were treated as IC_50_, representing the compound concentration that produces half-maximal inhibition, after confirming that these EC_50_ values reflected the calculated potency of inhibition. The collected data were carefully curated to extract those obtained under typical experimental conditions, specifically cell-based transport assays used to evaluate BCRP-mediated substrate transport and its inhibition by compounds. The details of the curation process are shown in Scheme S1, Supporting Information. After curation, IC_50_ values for 670 and 565 compounds were obtained from ChEMBL and Jiang et al., respectively. These data were merged by matching standardized SMILES. The final data set included 870 unique compounds, hereafter referred to as the BCRP data set.

To examine the variability in experimental IC_50_ values obtained under different conditions, the fold change between the maximum and minimum IC_50_ values was calculated. Among compounds with multiple IC_50_ records in ChEMBL, 96.7% (203 of 210) were within a 3-fold range, and 98.1% (358 of 363) in the overlapping compounds between the ChEMBL and Jiang et al. data sets were within a 3-fold range. Compounds showing a greater than 3-fold difference were manually checked using the original data. The experimental error of the IC_50_ values in the BCRP data set was therefore considered sufficiently small, owing to the careful curation of data obtained under unified experimental conditions. In addition, we constructed a data set of 4301 approved drugs and a list of 101 reported BCRP inhibitors, as described in the Experimental Section.

### Data Set Visualization

We visualized the BCRP data set using pIC_50_ values and drug–likeness parameters to characterize the acquired data. The distribution of pIC_50_ values is shown in Figure 1a, ranging from approximately 4 to 8, which correspond to IC_50_ values between 100 μM and 0.01 μM. In Figure 1b, the chemical space of the data set was compared with that of the approved drug data set using uniform manifold approximation and projection (UMAP). Although the approved drugs covered a broader area, the BCRP data set overlapped with partially populated regions of the approved drug space.

**Figure 1:**
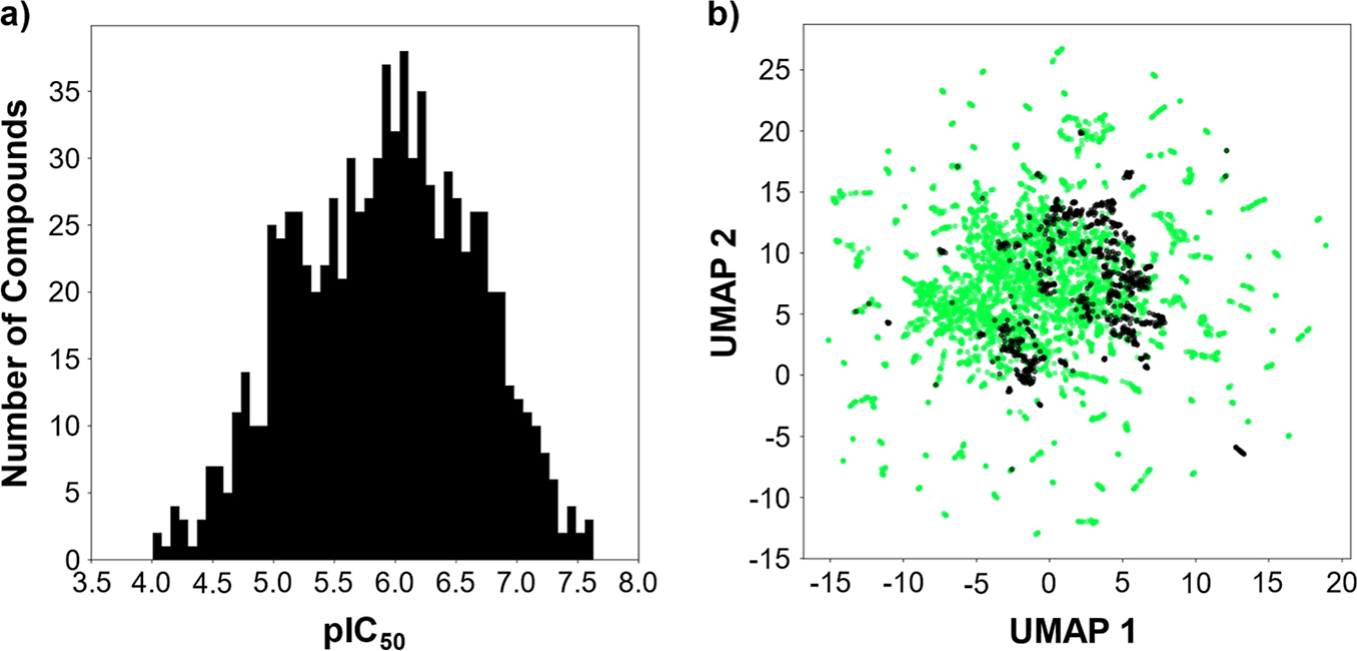
Data set visualization: (a) distribution of pIC_50_ values in the BCRP data set, (b) comparison of chemical space between compounds in the BCRP data set (black dots) and the data set of approved drugs (green dots).

We then focused on nine chemical properties, including the rule-of-five indicators that are likely to influence oral drug-likeness: molecular weight, topological polar surface area (TPSA), fraction of sp3 carbons, number of aromatic rings, number of hydrogen-bond acceptors, number of hydrogen-bond donors, number of heteroatoms, number of rotatable bonds, and octanol–water partition coefficient (Log *P*). The distributions of these nine parameters are shown in Figure 2. No significant bias was observed among these drug–likeness parameters, indicating that the data set includes a chemically diverse set of compounds.

**Figure 2:**
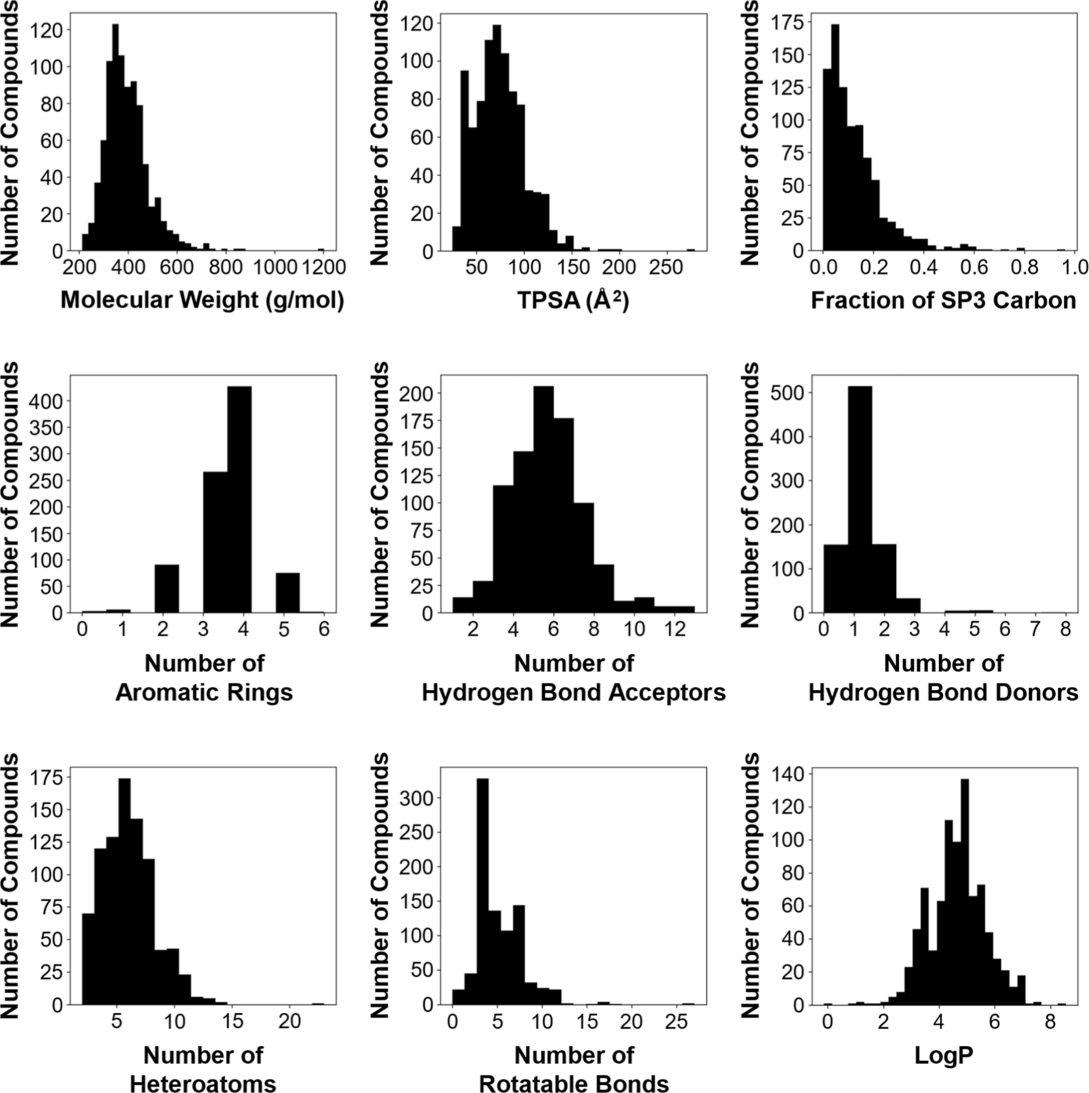
Distribution of compounds in the BCRP data set by representative drug-like parameters.

### Construction and Evaluation of Comprehensive Models

Regression models for predicting the inhibitory activity of BCRP were constructed using ML. After converting the chemical structure information into physicochemical descriptors and molecular fingerprints, the BCRP data set was randomly divided at a 9:1 ratio, and this process was repeated 10 times to avoid bias from random partitioning. To improve generalization performance by preventing the learning of unimportant feature patterns and reducing computation time, dimensionality reduction was applied to the features. Physicochemical descriptors and molecular fingerprints were treated separately during model construction using six algorithms (RF, LGBM, SVM, ANN, kNN, and RL), with hyperparameter optimization performed through Bayesian optimization. Model accuracy was evaluated using RMSE and *R* ^2^.

The results comparing the evaluation metrics for the two types of descriptors and six algorithms on the test sets are summarized in Table 1. Among them, LGBM showed the best performance, with *R* ^2^ values of 0.656 ± 0.065 and 0.574 ± 0.050, and RMSE values of 0.421 ± 0.035 and 0.476 ± 0.040 for the models using physicochemical descriptors and fingerprints, respectively. Models based on physicochemical descriptors tended to perform slightly better than those using molecular fingerprints.

**Table 1:** Evaluation Results of Models Using Different Algorithms and Feature Types on the Test Set

algorithm	evaluation metric	model with physicochemical descriptors	model with molecular fingerprints
RF	*R* ^2^	0.636 ± 0.065	0.542 ± 0.058
	RMSE	0.435 ± 0.042	0.494 ± 0.039
**LGBM**	***R*** ^2^	**0.656** **±** **0.065**	**0.574** **±** **0.050**
	**RMSE**	**0.421** **±** **0.035**	**0.476** **±** **0.040**
SVM	*R* ^2^	0.658 ± 0.069	0.544 ± 0.076
	RMSE	0.420 ± 0.045	0.490 ± 0.033
ANN	*R* ^2^	0.614 ± 0.076	0.485 ± 0.087
	RMSE	0.446 ± 0.045	0.522 ± 0.047
kNN	*R* ^2^	0.571 ± 0.070	0.459 ± 0.074
	RMSE	0.471 ± 0.047	0.537 ± 0.043
RL	*R* ^2^	0.570 ± 0.050	0.449 ± 0.080
	RMSE	0.473 ± 0.042	0.540 ± 0.031

The evaluation results of the best-performing models are shown in Figure 3 (left), with *R* ^2^ values of 0.736 and 0.649 and RMSE values of 0.386 and 0.465 for the physicochemical descriptor- and molecular fingerprint-based models, respectively. Additionally, 80.5% and 72.4% of the compounds were within a 3-fold error in the test set for the models using physicochemical descriptors and molecular fingerprints. The AUC-ROC curves with various threshold values were plotted to demonstrate the possible use of the regression models as classification models when the thresholds were varied between pIC_50_ = 5 and 7. AUC-ROC values were approximately 0.9, indicating strong discriminative performance across a wide range of decision thresholds, as shown in Figure 3 (right). Therefore, we successfully constructed predictive models for the inhibitory activity of BCRP based on chemical structure information.

**Figure 3:**
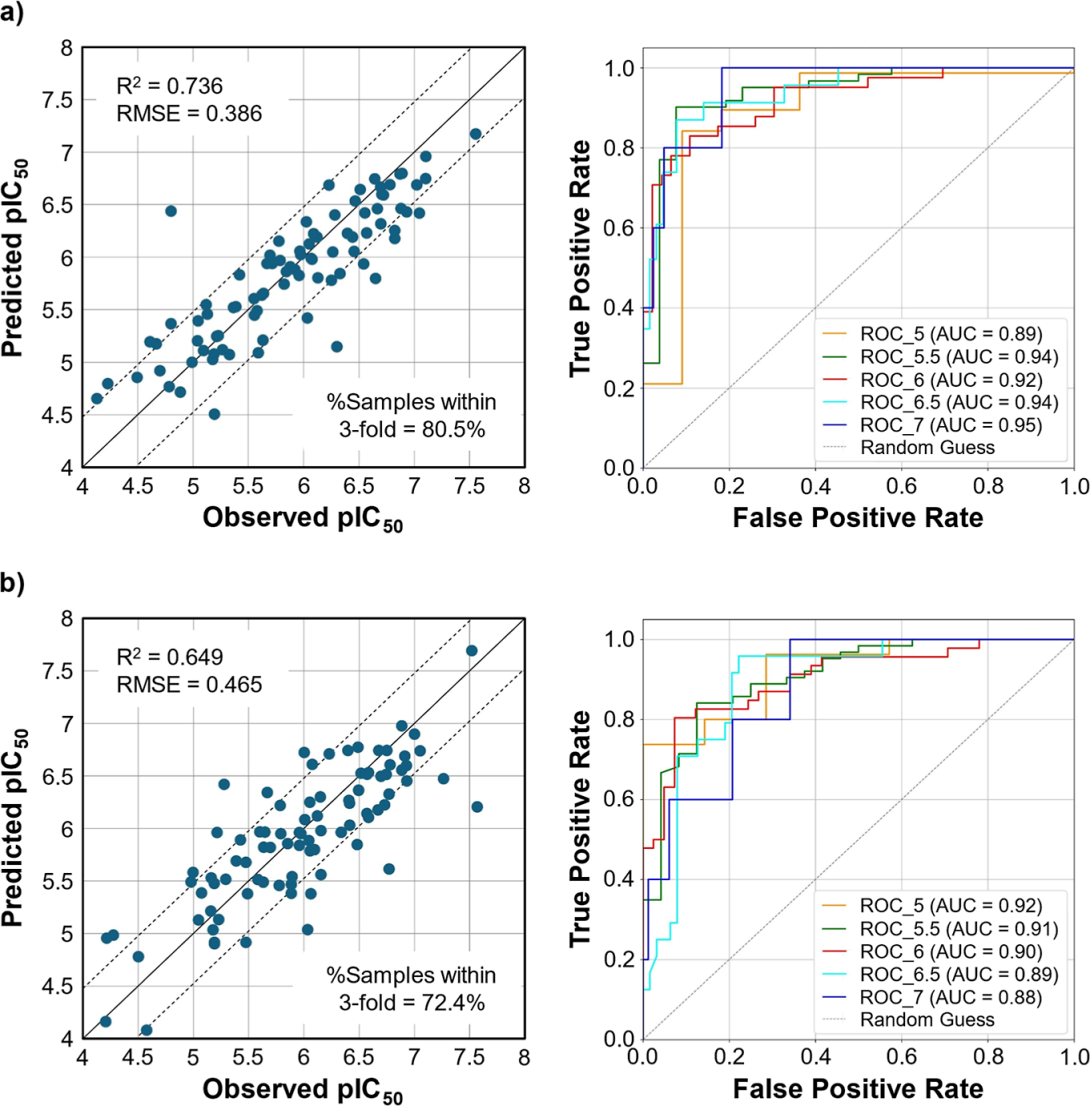
Evaluation results of the best-performing model using LGBM, with (a) physicochemical properties and (b) molecular fingerprints as features on the test set. Left: plots of observed vs predicted pIC_50_; Right: AUC-ROC curves at thresholds of 5, 5.5, 6, 6.5, and 7.

To interpret the model predictions, we focused on the fingerprint generation process. Some of the fingerprints used in the best model, such as RAD2D and DFS, involve hashing, which is an irreversible operation. In such cases, it is difficult to convert the binary vectors back into their corresponding substructures, making it challenging to evaluate the contribution of each substructure in a compound to the predicted value. Although the predictive performance of the constructed model was sufficient, there remains considerable potential for improvement in its interpretability.

### Construction and Evaluation of Simplified Descriptor Models

To address the difficulty in interpreting the predicted results, a simplified descriptor model was constructed by restricting the descriptors to substructure-based fingerprints (MACCS Keys, PubChem Fingerprint, and Klekota-Roth Fingerprint calculated using PaDEL-Descriptor) and the algorithms to tree-based or linear models (RF, LGBM, and RL). To construct the simplified descriptor model, further feature reduction was performed to identify the minimum number of features required for a simple yet effective predictive model. After applying Boruta_py for feature selection, additional reduction was gradually carried out using RFE while monitoring the changes in *R* ^2^ and RMSE values. We found that *R* ^2^ and RMSE began to deteriorate when the number of features decreased below 170; therefore, we constructed our second model using these 170 features (Figure S3).

Prediction models were then built with the selected 170 features. The prediction results for the same test sets as the comprehensive models are shown in Table 2. The RF model achieved the best performance, with *R* ^2^ and RMSE values of 0.629 ± 0.059 and 0.443 ± 0.029, respectively, which were slightly better than those of the initial comprehensive model using molecular fingerprints as features. Figure 4 presents the evaluation results of the best-performing fold of the RF model on the test set. The model showed an *R* ^2^ of 0.703 and an RMSE of 0.430, with 75.9% of compounds within a 3-fold error in the test set. The AUC-ROC values were approximately 0.9 when thresholds were varied between pIC_50_ = 5 and 7.

**Table 2:** Evaluation Results of Simplified Models Using Selected 170 Substructure-Based Fingerprint Features with Different Algorithms on the Test Set

	evaluation metric	
algorithm	*R* ^2^	RMSE
RF	0.629 ± 0.059	0.443 ± 0.029
LGBM	0.624 ± 0.053	0.446 ± 0.030
RL	0.531 ± 0.064	0.500 ± 0.047

**Figure 4:**
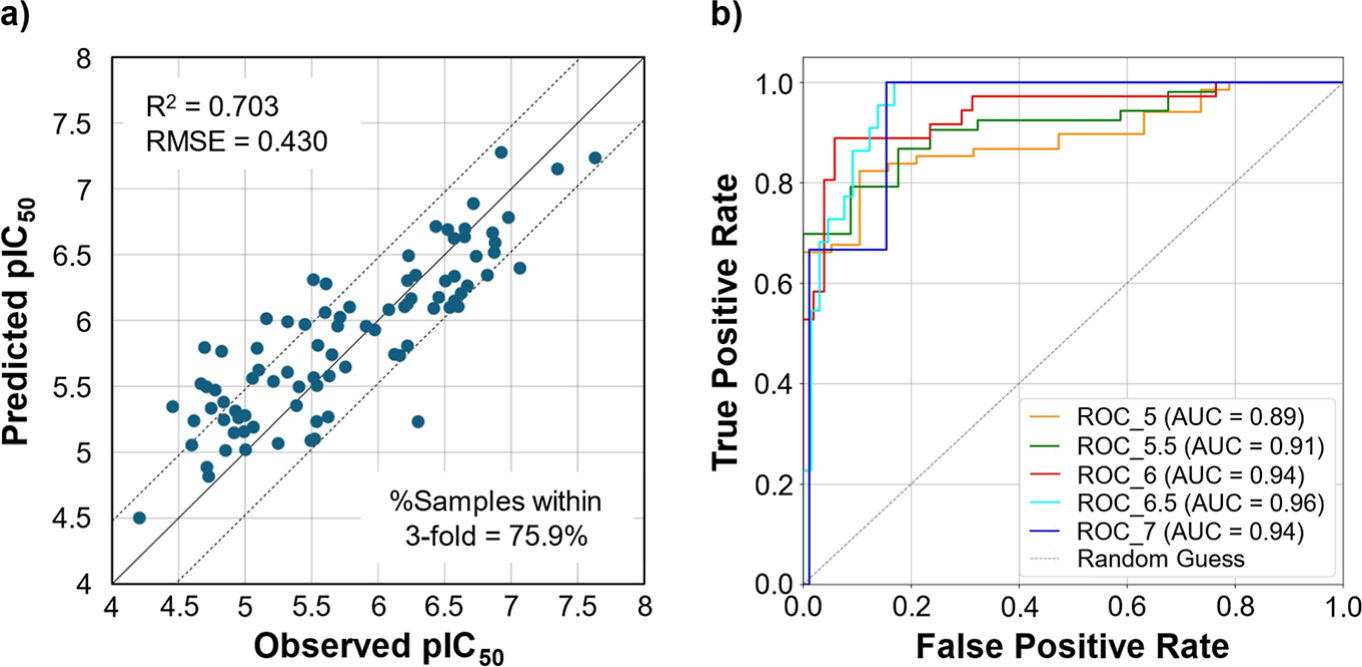
Evaluation results of the best-performing model using RF with selected 170 molecular fingerprints as features on the test set: (a) plot of observed vs predicted pIC_50_ and (b) AUC-ROC curve at thresholds of 5, 5.5, 6, 6.5, and 7.

To make the most effective use of the available data, we retrained the RF model using all data with the same 170 features, following the same procedure as before, and finalized it. This model is hereafter referred to as the finalized model.

### Explainability of the Finalized Model

SHAP analysis was conducted to interpret the predictions of the finalized model. Figure 5a shows the most important features identified in the simplified descriptor model based on SHAP analysis. The most important features appeared to be positively correlated with the predicted inhibitory activity. Accordingly, the presence of substructures corresponding to these important features contributes to higher predicted inhibitory activity. The top five features were PubChem Fingerprint 359, PubChem Fingerprint 576, MACCS Fingerprint 151, PubChem Fingerprint 821, and PubChem Fingerprint 577, and their corresponding substructures are shown in Figure 5b.

**Figure 5:**
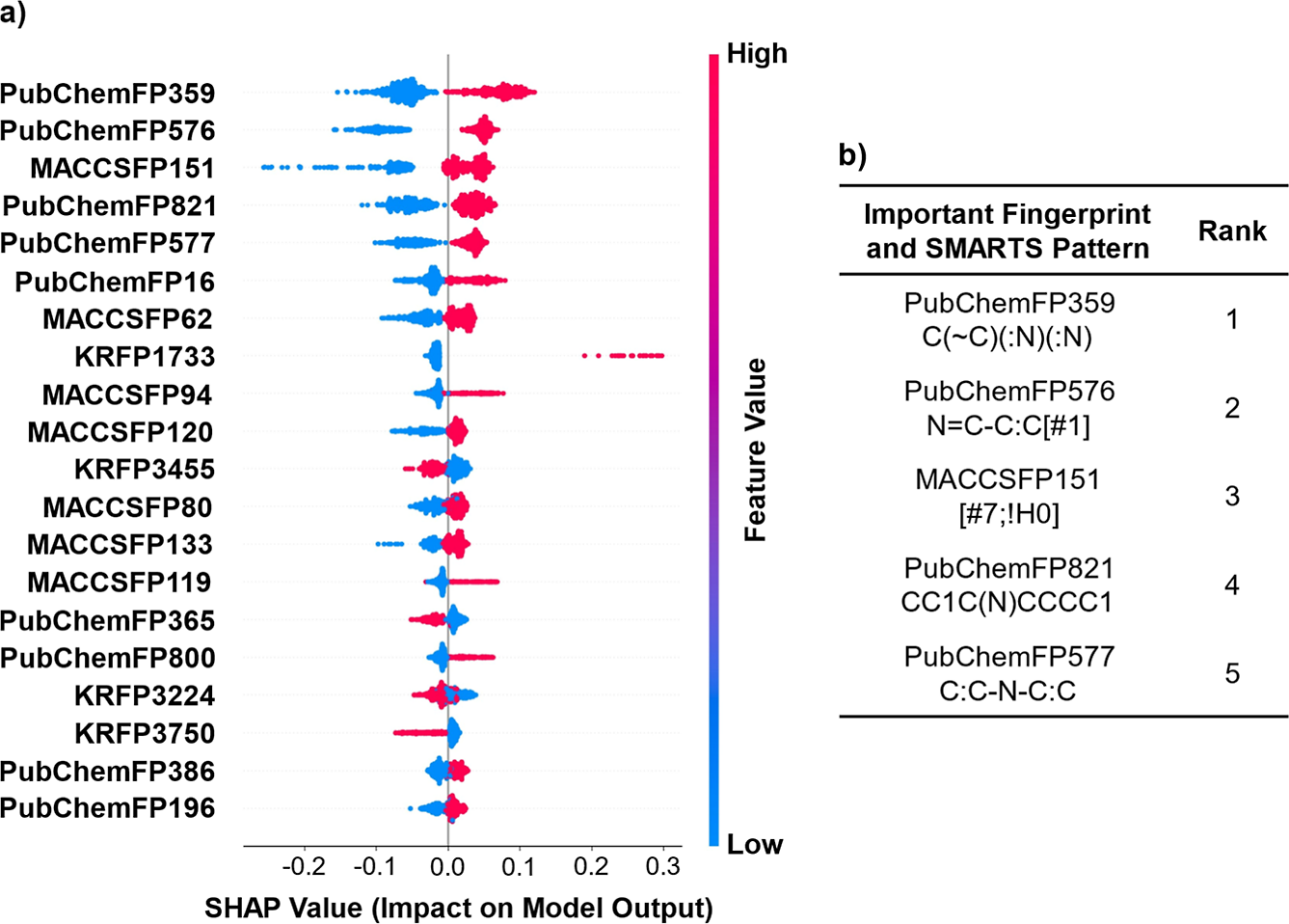
(a) SHAP summary plot of the finalized model. (b) Structural information on the top five important features.

As an additional method to assess feature importance, the ranking of important features was also obtained from the RF model. The top five features were consistent with the SHAP results, and many of the same features appeared within the top 20, indicating a strong agreement between the two approaches. The list of important features obtained from the RF model is shown in Table S4.

Among the substructures identified in our study based on SHAP analysis, the presence of nitrogen atoms (PubChemFP16), patterns commonly found in heteroaromatic or heterocyclic rings (MACCSFP80, Heterocyclic atom >1), a disubstituted heteroaromatic ring containing two nitrogen atoms (PubChemFP359), and other substructural features consistent with previous studies were identified. In addition, a nonaromatic ring structure containing heteroatoms (PubChemFP800, PubChemFP821), and a benzene ring substituted with a nitro group and at least one additional substituent (KRFP1733), which has not been reported in previous studies, were identified as important features for prediction. These substructures showed high SHAP contributions and represented chemically interpretable motifs among the top-ranked features. A list of these substructures is presented in Table 3.

**Table 3:**
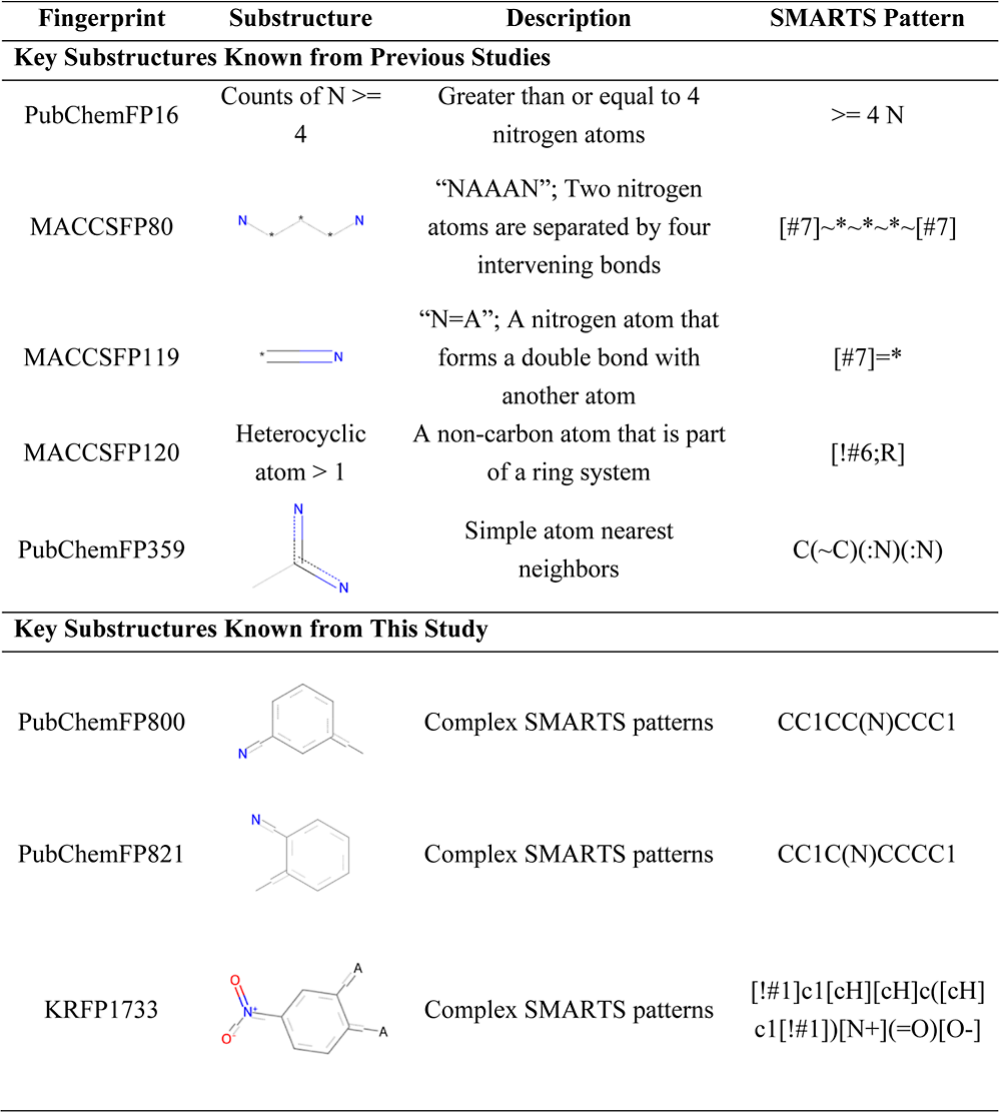
Examples of Key Substructures Associated with Predictive Inhibitory Activity in Comparison with Previous Studies

We performed a Student’s *t*-test to compare the means of two groups and a Kolmogorov–Smirnov (KS) test to compare their distributions, with or without the presence of these eight substructures. All *p*-values obtained from both the *t*-test and KS-test were less than 0.01, indicating a statistically significant difference in pIC_50_ values between the two groups. The distributions of pIC_50_ values for the two groups with each fingerprint are shown in Figure S4.

### Potential BCRP Inhibitors in Approved Drugs

With the aim of identifying approved drugs that potentially inhibit BCRP, we predicted the inhibitory activity of 4301 approved drugs, excluding 18 compounds that overlapped with the BCRP data set, using the finalized model. When the chemical space of an external data set is much broader than that of the training data set, prediction accuracy outside the model’s applicability domain may decrease,^42^ leading to an increased FPR when applied to randomly selected compounds. To minimize unreliable predictions, we set a cutoff value based on structural similarity to the compounds used for training.

For the 10 different test sets used during the validation of the simplified descriptor model, the max Tanimoto between each test compound and the compounds in the corresponding training set was calculated, and the compound groups classified as TP and FP were identified. Subsequently, cutoff values were determined at the points where the FPR fell below 0.3. FPR was selected as the primary criterion because false positives have the greatest impact on screening efficiency in early stage drug discovery. The average cutoff value obtained from the 10 test sets, 0.691, was adopted as the final cutoff value. The average FPR corresponding to this threshold was 0.223. The plots used to determine the cutoff values are shown in Figure S2.

Next, the max Tanimoto values for the approved drug data set were determined, and the final cutoff value was applied. Approved drugs were then classified into high (max Tanimoto >0.691) and low (max Tanimoto ≤ 0.691) similarity groups relative to the BCRP data set. To characterize chemical features of compounds outside this similarity-based applicability domain, selected molecular descriptors were compared between these two groups to identify chemical classes that may fall outside the model’s reliable predictive range. Figure S5 suggests that compounds with descriptor values outside the following ranges may lie beyond the applicability domain: average molecular weight 248.25–1214.65 g/mol, *S* log *P* – 0.14–6.31, TPSA 37.3–278.8 Å^2^, number of heavy atoms 19–86, number of saturated rings 0–3, and number of aliphatic heterocycles 0, 1, or 3.

As shown in Table 4, 19 compounds exhibited a max Tanimoto >0.691. Among these, seven compounds were previously reported as BCRP inhibitors, six of which showed pIC_50_ > 5, indicating that our model successfully reproduced previous findings. In addition, 12 approved drugs with max Tanimoto >0.691 were not previously reported as BCRP inhibitors, among which seven compounds (benziodarone, rosuvastatin, atorvastatin, axitinib, pimecrolimus, tacrolimus, and voclosporin) were predicted to have pIC_50_ > 5, as shown in Table 4.

**Table 4:** Prediction of pIC_50_ Values of 19 Approved Drugs with Maximum Tanimoto Similarity >0.691 to the Training Set

ChEMBL ID	KEGG ID	compound name	predicted pIC_50_
CHEMBL232201	D01833	Benziodarone	5.901
CHEMBL411	D00577	Diethylstilbestrol^a^	5.810
CHEMBL553	DG00711	Erlotinib^a^	5.711
CHEMBL1496	D08492	Rosuvastatin	5.561
CHEMBL1487	D07474	Atorvastatin	5.470
CHEMBL1289926	D03218	Axitinib	5.469
CHEMBL1405	D00067	Estrone^a^	5.330
CHEMBL1200686	D05480	Pimecrolimus	5.321
CHEMBL3989887	D00107	Tacrolimus	5.315
CHEMBL160	D00184	Cyclosporine^a^	5.312
CHEMBL5314379	D09033	Voclosporin	5.312
CHEMBL1200430	D04061	Estradiol acetate^a^	5.137
CHEMBL114	D00429	Saquinavir^a^	5.003
CHEMBL163	D00427	Ritonavir^a^	4.982
CHEMBL584	D08259	Nelfinavir	4.952
CHEMBL53463	D03899	Doxorubicin	4.666
CHEMBL36506	D01209	Novobiocin	4.599
CHEMBL1117	D08062	Idarubicin	4.570
CHEMBL178	D07776	Daunorubicin	4.531

a Compound previously reported as a BCRP inhibitor.

## Discussion

In this study, in silico prediction models for BCRP inhibitory activity were constructed. Comprehensive models with sufficient predictive accuracy, along with feature simplification, improved the simplicity of the prediction results. In addition, seven potential BCRP inhibitors were identified among approved drugs based on the prediction results.

The advantages of the regression model developed for predicting BCRP inhibition include its flexibility in threshold settings by directly predicting numerical values, allowing expansion of its application range according to the research objective. The model can be used for various purposes, including classification and ranking. It is crucial to minimize discrepancies between predicted results and actual measurements to ensure the reliability of regression-based predictions. Regarding data quality, the collected data were carefully curated to include only those obtained under typical experimental conditions, specifically cell-based transport assays that evaluate BCRP-mediated substrate transport and its inhibition by compounds. The error range of the curated data was sufficiently small; therefore, we believe that the data set provided is of high quality.

Regarding the model performance, the evaluation results of all the best models showed that more than 70% of compounds in the test set were within a 3-fold error, and the AUC-ROC values were approximately 0.9 when the thresholds were shifted between pIC_50_ = 5 and 7. These results indicate that the constructed models successfully predict the inhibitory activity of BCRP. Furthermore, we computed metrics by converting regression predictions into classification outputs and compared them with previously reported studies using pIC_50_ = 5 (IC_50_ = 10 μM) as the threshold value. Although different pIC_50_ criteria are set depending on the study stage or analytical purpose, an IC_50_ value of 10 μM (pIC_50_ = 5) is generally used as a threshold to classify inhibitors and noninhibitors.^43,44^ Previous studies have reported MCC values of 0.812 ^12^ and 0.63,^13^ and BA values of 0.905 ^12^ and 0.82 ^13^ for BCRP inhibition prediction models. In comparison, the best simplified descriptor model developed in this study achieved an MCC of 0.68 and a BA of 0.75 (Table S5). Although direct comparison is not possible because of different test sets, these results suggest that our model performs comparably to previously reported classification models.

Although the regression outputs were converted into binary classes to enable comparison with previous classification-based studies, it should be noted that the model was primarily optimized for continuous pIC_50_ prediction. The resulting BA of 0.75 therefore reflects a deliberate trade-off, as the regression framework prioritizes numerical ranking and compound prioritization rather than binary decision-making. This capability provides greater practical utility for virtual screening, while still maintaining competitive classification performance.

Previous studies on BCRP inhibition have reported the following findings. Matsson et al.^45,46^ noted that the octanol–water partition coefficient at pH 7.4 (log *D* 7.4), the number of nitrogen atoms, the presence of C–S bonds, molecular planarity, and aromaticity influence inhibitor binding to BCRP. They also stated that the presence of a sulfate group is unfavorable for BCRP inhibition because it is bulky and may cause steric hindrance at the binding site. Jiang et al.^12^ further reported that BCRP inhibitors tend to have more hydrogen bond acceptors and fewer hydrogen bond donors than noninhibitors. In addition, Sjostedt et al.^47^ observed that BCRP inhibitors generally contain more rings than noninhibitors. Moreover, Moinul et al.^48^ suggested that potential structural features of BCRP inhibitors include the fused ring of benzene with a six-member heterocyclic aromatic ring, disubstituted heterocyclic rings, methoxy phenyl rings, and amino, carbonyl, or amide groups as linkers.

By analyzing the SHAP values of the finalized models, we successfully identified important substructures for predicting BCRP inhibition (Table 3). The previously reported characteristics and substructures were consistent with those extracted by our model, particularly heteroaromatic or heterocyclic rings containing nitrogen, indicating that the constructed model can reliably capture compound properties and substructures relevant to BCRP inhibition. Furthermore, we identified novel substructures (PubChemFP800, PubChemFP821, and KRFP1733) that have not been reported previously, which may contribute to the design of new BCRP inhibitors.

Although direct experimental validation of the proposed interaction mechanisms is currently unavailable, the interpretations are consistent with previous studies on BCRP inhibition. Prior reports have shown that BCRP inhibitors are generally characterized by high aromaticity, increased hydrophobicity, molecular planarity, and a higher number of hydrogen bond acceptors with fewer hydrogen bond donors.^12,46,47^ In addition, substituted aromatic and heterocyclic ring systems have been repeatedly implicated as key structural motifs in BCRP inhibitors.^48^

In this context, the nitro-substituted aromatic ring identified in our analysis aligns well with these established trends. Aromatic rings may contribute to BCRP inhibition through π–π stacking or hydrophobic interactions within the relatively large and flexible substrate-binding cavity of BCRP, while the nitro substituent, as a strong electron-withdrawing and hydrogen-bond-accepting group, may modulate the electronic properties of the aromatic system and enhance interactions with polar residues in the binding pocket. Taken together, these considerations indicate that the newly identified substructures are not contradictory to previous findings but rather represent a substructure-level refinement and extension of known physicochemical and structural determinants of BCRP inhibition.

Although distribution bias cannot be completely avoided due to the use of data from public databases and literature, we expect that incorporating additional experimental data covering a wider range of chemical structures, especially those with higher and lower pIC_50_ values, will further improve the generalizability of the model.

We constructed two types of models: a comprehensive model and a simplified descriptor model. The comprehensive model serves as a baseline, incorporating a wide range of feature representations. This approach is common in predictive modeling and yields high accuracy (Figure 3) but limited interpretability. Therefore, the comprehensive model is preferable when prediction accuracy is the primary goal and interpretability is less critical. In contrast, the simplified descriptor model, which employs structure-based fingerprints, offers greater interpretability while maintaining competitive performance (Figure 4). Including both models allows us to assess the trade-offs between accuracy and interpretability, providing a more holistic perspective on model selection.

In 2012, the FDA draft guidance for DDI studies encouraged the involvement of intestinal BCRP when evaluating drug transporters.^8^ Therefore, some approved drugs marketed before 2012 may not have been examined for their BCRP inhibitory activity. To identify such compounds, we predicted the inhibitory activity of approved drugs using our BCRP model.

Our data set consists of compounds with pIC_50_ values ranging from 4 to 8, of which approximately 10% (94 of 870) have pIC_50_ < 5, generally considered as noninhibitors. This distribution likely reflects the tendency of in vitro screening assays to exclude notable noninhibitors, as IC_50_ values are typically calculated only for active compounds. Consequently, when applying the model to random compound sets, the FPR may increase. This issue arises when using a model built on the BCRP data set to predict approved drugs, whose chemical space is much broader than that of our training data (Figure 1). Because prediction accuracy in machine learning decreases when the test data differ from the training data, many overestimated prediction values (FP) can occur. To address this, we introduced a cutoff based on the Tanimoto coefficient and applied it to the approved drugs.

The final cutoff was determined according to the FPR by distinguishing TP and FP in the test sets. The acceptable range of FPR varies depending on the screening purpose and target characteristics, making a universal threshold difficult to define. However, previous studies on predictive models for screening have reported FPR values between 0.1 and 0.8, with approximately 0.3 considered typical.^49,50^ Therefore, we set an FPR threshold below 0.3 to achieve an appropriate balance. Since the final averaged FPR was 0.22, we expect that applying the Tanimoto cutoff to the external test set will yield an FPR of around 0.22.

As a result of applying the model to the data set of approved drugs, 18 compounds were filtered, and seven compounds newly showed potential as BCRP inhibitors. Although tacrolimus and voclosporin were not included in the list of reported BCRP inhibitors, they have been previously reported to exhibit inhibitory activity,^51,52^ indicating that our model was able to identify known BCRP inhibitors. In addition, rosuvastatin, atorvastatin, and axitinib are known BCRP substrates,^53–55^ suggesting that these compounds may act as inhibitors via competitive inhibition. Benziodarone and pimecrolimus were predicted by our model as BCRP inhibitors, although no prior reports have indicated any relationship with BCRP; thus, experimental verification is required.

Currently, the amount of publicly available data on BCRP inhibition is limited, and potential biases exist in the reported IC_50_ values. Therefore, it is essential to continue collecting more data, re-evaluating the model, and updating it with novel data. These efforts will expand the model’s applicability domain and enable more reliable predictions for a larger number of compounds across a broader chemical space.

## Conclusions

We constructed a high-quality BCRP data set comprising 870 compounds and developed a comprehensive in silico regression model to predict the pIC_50_ values of compounds against BCRP with sufficient accuracy. To improve interpretability, a simplified descriptor model was also constructed, which achieved comparable accuracy to the comprehensive model. In addition, we identified novel substructures that contribute to BCRP inhibition, along with substructures previously reported in the literature. Furthermore, more than 4000 approved drugs were screened, among which seven were identified as potential BCRP inhibitors. This model can be applied during the early stages of drug development to provide a preliminary estimation of a compound’s ADMET properties prior to in vitro experiments. In the future, as more data become available in underrepresented chemical spaces, models with improved generalization performance can be developed. These models contribute to accelerating and reducing the cost of compound screening during early drug discovery, while also helping to exclude potential future drop-out candidates.

## Supplementary Material



## Data Availability

The data and code used in this paper are available on GitHub at https://github.com/rw-1746/BI_pred

## Notes

Table S1: List of descriptors used in the study; Table S2: Optuna settings for the comprehensive model; Table S3: Optuna settings for the simplified descriptor model; Table S4: Top 20 important features in the simplified descriptor model (RF); Table S5: Comparison of converted classification metrics with previous studies; Scheme S1: Details of the manual curation process for the BCRP data set; Figure S1: Overview of machine learning process; Figure S2: Determination of the cutoff based on the Tanimoto coefficient using the 10 test sets of the simplified descriptor model; Figure S3: Observed *R* ^2^, RMSE, and MSE during feature reduction by RFE; Figure S4: Distribution of pIC_50_ values in groups with or without important fingerprints; Figure S5: Comparison of selected molecular descriptors between approved drugs with high and low structural similarity to the BCRP data set (PDF)

## Abbreviations

ABCG2: ATP-binding cassette subfamily G member 2
ADMET: absorption, distribution, metabolism, excretion, and toxicity
AI: artificial intelligence
ANN: artificial neural network
ATP: adenosine triphosphate
AUC: area under curve
BA: balanced accuracy
BBB: blood–brain barrier
BCRP: breast cancer-resistance protein
CNS: central nervous system
DDI: drug–drug interaction
DFS: depth first search
EC_50_: 50% effective concentration
EMA: European medicines agency
FDA: U.S. food and drug administration
FN: false negative
FP: false positive
FP: fingerprint
FPR: false positive rate
IC_50_: 50% inhibition concentration
KEGG: Kyoto encyclopedia of genes and genomes
kNN: k-nearest neighbor
KR: Klekota-Roth
KS: Kolmogorov–Smirnov
LGBM: light gradient boosting machine
MACCS: molecular access system
MCC: Matthew’s correlation coefficient
ML: machine learning
PCA: principal component analysis
QSAR: quantitative structure–activity relationship
RF: random forest
RFE: recursive feature elimination
RL: Ridge and Lasso
SHAP: Shapley additive explanation
SMARTS: SMILES arbitrary target specification
SMILES: simplified molecular input line entry system
SVM: support vector machine
TN: true negative
TP: true positive
TPR: true positive rate
TPSA: topological polar surface area
UMAP: uniform manifold approximation and projection

## References

[1] Bai F., Li S., Li H. (2024). AI Enhances Drug Discovery and Development. Natl. Sci. Rev..

[2] Ferreira L.
L. G., Andricopulo A. D. (2019). ADMET Modeling
Approaches in Drug
Discovery. Drug Discovery Today.

[3] Venkatraman V. (2021). FP-ADMET:
A Compendium of Fingerprint-Based ADMET Prediction Models. J. Cheminf..

[4] Ogbonna U. E., Ofoezie E. F., Babalola O. O., Ottu P. O., Ogbonna C. A., Olisakwe S., George T. E., Babarinde S., Omaba J. O., Chukwuemeka C. G., Amafili C. C., Afamefuna A., Ogbonna H. (2025). Advances in Machine
Learning for Optimizing Pharmaceutical
Drug Discovery. Curr. Proteomics.

[5] Yi J., Shi S., Fu L., Yang Z., Nie P., Lu A., Wu C., Deng Y., Hsieh C., Zeng X., Hou T., Cao D. (2024). OptADMET: A Web-Based Tool for Substructure Modifications to Improve
ADMET Properties of Lead Compounds. Nat. Protoc..

[6] Engdahl E., van Schijndel M. D. M., Voulgaris D., Di Criscio M., Ramsbottom K. A., Rigden D. J., Herland A., Rüegg J. (2021). Bisphenol
A Inhibits the Transporter Function of the Blood-Brain Barrier by
Directly Interacting with the ABC Transporter Breast Cancer Resistance
Protein (BCRP). Int. J. Mol. Sci..

[7] Strazielle N., Ghersi-Egea J.-F. (2015). Efflux Transporters in Blood-Brain Interfaces of the
Developing Brain. Front. Neurosci..

[8] Center for Drug Evaluation and Research Guidance for Industry. http://www.fda.gov/Drugs/GuidanceComplianceRegulatoryInformation/Guidances/default.htm (accessed 2024–05–27).

[9] Liu, X. Transporter-Mediated Drug-Drug Interactions and Their Significance. In Drug Transporters in Drug Disposition, Effects and Toxicity; Liu, X. , Pan, G. , Eds.; Springer Singapore: Singapore, 2019; pp 241–291. 10.1007/978-981-13-7647-4_5. 31571167

[10] Administration, U. S. F. ; Clinical, D. Drug Interaction Studies-Cytochrome P450 Enzyme and Transporter-Mediated Drug Interactions Guidance for Industry, 2020.

[11] Cole S., Kerwash E., Andersson A. (2020). A Summary of the Current Drug Interaction
Guidance from the European Medicines Agency and Considerations of
Future Updates. Drug Metab. Pharmacokinet..

[12] Jiang D., Lei T., Wang Z., Shen C., Cao D., Hou T. (2020). ADMET Evaluation
in Drug Discovery. 20. Prediction of Breast Cancer Resistance Protein
Inhibition through Machine Learning. J. Cheminf..

[13] Huang S., Gao Y., Zhang X., Lu J., Wei J., Mei H., Xing J., Pan X. (2022). Development
of Simple and Accurate
in Silico Ligand-Based Models for Predicting ABCG2 Inhibition. Front. Chem..

[14] Kong X., Lin K., Wu G., Tao X., Zhai X., Lv L., Dong D., Zhu Y., Yang S. (2023). Machine Learning Techniques
Applied to the Study of Drug Transporters. Molecules.

[15] Ding Y.-L., Shih Y.-H., Tsai F.-Y., Leong M. K. (2014). In Silico Prediction
of Inhibition of Promiscuous Breast Cancer Resistance Protein (BCRP/ABCG2). PLoS One.

[16] Bhatia N., Thareja S. S. (2025). 3D-QSAR, Molecular Docking and Pharmacophore
Mapping
Studies on Indole Derivatives as Aromatase Inhibitors. Lett. Drug Des. Discovery.

[17] Glen R. C., Cole J. C., Stewart J. J. P. (2025). Prediction of
Enzyme Inhibition (IC50)
Using a Combination of Protein–Ligand Docking and Semiempirical
Quantum Mechanics. J. Mol. Model..

[18] Mao J., Akhtar J., Zhang X., Sun L., Guan S., Li X., Chen G., Liu J., Jeon H.-N., Kim M. S., No K. T., Wang G. (2021). Comprehensive Strategies of Machine-Learning-Based
Quantitative Structure-Activity Relationship Models. iScience.

[19] Sheu R.-K., Pardeshi M. S. (2022). A Survey on Medical
Explainable AI (XAI): Recent Progress,
Explainability Approach, Human Interaction and Scoring System. Sensors.

[20] Mendez D., Gaulton A., Bento A. P., Chambers J., De Veij M., Félix E., Magariños M. P., Mosquera J. F., Mutowo P., Nowotka M., Gordillo-Marañón M., Hunter F., Junco L., Mugumbate G., Rodriguez-Lopez M., Atkinson F., Bosc N., Radoux C. J., Segura-Cabrera A., Hersey A., Leach A. R. (2019). ChEMBL:
Towards
Direct Deposition of Bioassay Data. Nucleic
Acids Res..

[21] Kanehisa M., Furumichi M., Sato Y., Kawashima M., Ishiguro-Watanabe M. (2023). KEGG for Taxonomy-Based Analysis of Pathways and Genomes. Nucleic Acids Res..

[22] PubChemPy PubChemPy 1.0.5, 2024. https://pubchempy.readthedocs.io/en/latest/#.

[23] Knox C., Wilson M., Klinger C. M., Franklin M., Oler E., Wilson A., Pon A., Cox J., Chin N. E., Strawbridge S., Garcia-Patino M., Kruger R., Sivakumaran A., Sanford S., Doshi R., Khetarpal N., Fatokun O., Doucet D., Zubkowski A., Rayat D., Jackson H., Harford K., Anjum A., Zakir M., Wang F., Tian S., Lee B., Liigand J., Peters H., Wang R. Q., Nguyen T., So D., Sharp M., da Silva R., Gabriel C., Scantlebury J., Jasinski M., Ackerman D., Jewison T., Sajed T., Gautam V., Wishart D., Gautam V., Wishart D. S. (2024). DrugBank
6.0: The DrugBank Knowledgebase for 2024. Nucleic
Acids Res..

[24] Heyndrickx W., Mervin L., Morawietz T., Sturm N., Friedrich L., Zalewski A., Pentina A., Humbeck L., Oldenhof M., Niwayama R., Schmidtke P., Fechner N., Simm J., Arany A., Drizard N., Jabal R., Afanasyeva A., Loeb R., Verma S., Harnqvist S., Holmes M., Pejo B., Telenczuk M., Holway N., Dieckmann A., Rieke N., Zumsande F., Clevert D.-A., Krug M., Luscombe C., Green D., Ertl P., Antal P., Marcus D., Do Huu N., Fuji H., Pickett S., Acs G., Boniface E., Beck B., Sun Y., Gohier A., Rippmann F., Engkvist O., Göller A. H., Moreau Y., Galtier M. N., Schuffenhauer A., Ceulemans H. (2024). MELLODDY: Cross-Pharma Federated
Learning at Unprecedented Scale Unlocks Benefits in QSAR without Compromising
Proprietary Information. J. Chem. Inf. Model..

[25] RDKit RDKit: Open-Source Cheminformatics Software, 2024. https://www.rdkit.org.

[26] Moriwaki H., Tian Y.-S., Kawashita N., Takagi T. (2018). Mordred: A Molecular
Descriptor Calculator. J. Cheminf..

[27] Yap C. W. (2011). PaDEL-Descriptor:
An Open Source Software to Calculate Molecular Descriptors and Fingerprints. J. Comput. Chem..

[28] Hinselmann G., Rosenbaum L., Jahn A., Fechner N., Zell A. (2011). JCompoundMapper:
An Open Source Java Library and Command-Line Tool for Chemical Fingerprints. J. Cheminf..

[29] Pedregosa F., Varoquaux G., Gramfort A., Michel V., Thirion B., Grisel O., Blondel M., Prettenhofer P., Weiss R., Dubourg V., Vanderplas J., Passos A., Cournapeau D., Brucher M., Perrot M., Duchesnay E. ´. (2011). Scikit-Learn:
Machine Learning in Python. J. Mach. Learn.
Res..

[30] McInnes L., Healy J., Saul N., Großberger L. (2018). UMAP: Uniform
Manifold Approximation and Projection. J. Open
Source Softw..

[31] Kursa M. B., Rudnicki W. R. (2010). Feature Selection
with the Boruta Package. J. Stat. Softw..

[32] Breiman L. (2001). Random Forests. Mach. Learn..

[33] Akiba, T. ; Sano, S. ; Yanase, T. ; Ohta, T. ; Koyama, M. Optuna: A Next-Generation Hyperparameter Optimization Framework. In Proceedings of the 25th ACM SIGKDD International Conference on Knowledge Discovery & Data Mining; KDD ’19; Association for Computing Machinery: New York, NY, USA, 2019; pp 2623–2631. 10.1145/3292500.3330701.

[34] Ke, G. ; Meng, Q. ; Finley, T. ; Wang, T. ; Chen, W. ; Ma, W. ; Ye, Q. ; Liu, T.-Y. LightGBM: A Highly Efficient Gradient Boosting Decision Tree. In Proceedings of the 31st International Conference on Neural Information Processing Systems; NIPS’17; Curran Associates Inc.: Red Hook, NY, USA, 2017; pp 3149–3157.

[35] Cortes C., Vapnik V. (1995). Support-Vector Networks. Mach.
Learn..

[36] Rumelhart D. E., Hinton G. E., Williams R. J. (1986). Learning
Representations by Back-Propagating
Errors. Nature.

[37] Knee Finder Knee Finder, 2022. https://github.com/vlavorini/kneefinder.

[38] SHAP SHAP Documentation, 2018. https://shap.readthedocs.io/en/latest/index.html.

[39] Kim S., Chen J., Cheng T., Gindulyte A., He J., He S., Li Q., Shoemaker B. A., Thiessen P. A., Yu B., Zaslavsky L., Zhang J., Bolton E. E. (2023). PubChem 2023 Update. Nucleic Acids Res..

[40] National Center for Biotechnology Information PubChem: Substructure Fingerprint V1.3; National Center for Biotechnology Information, 2009..

[41] RDKit RDKit MACCS Implementation, 2023. https://github.com/rdkit/rdkit/blob/master/rdkit/Chem/MACCSkeys.py.

[42] Sahigara F., Mansouri K., Ballabio D., Mauri A., Consonni V., Todeschini R. (2012). Comparison
of Different Approaches to Define the Applicability
Domain of QSAR Models. Molecules.

[43] Huang S.-M., Zhang L., Giacomini K. M. (2010). The International
Transporter Consortium:
A Collaborative Group of Scientists From Academia, Industry, and the
FDA. Clin. Pharmacol. Ther..

[44] Shoemaker R. H. (2006). The NCI60
Human Tumour Cell Line Anticancer Drug Screen. Nat. Rev. Cancer.

[45] Matsson P., Englund G., Ahlin G., Bergström C. A. S., Norinder U., Artursson P. (2007). A Global Drug Inhibition Pattern
for the Human ATP-Binding Cassette Transporter Breast Cancer Resistance
Protein (ABCG2)^s^. J. Pharmacol. Exp.
Ther..

[46] Matsson P., Pedersen J. M., Norinder U., Bergström C. A. S., Artursson P. (2009). Identification
of Novel Specific and General Inhibitors
of the Three Major Human ATP-Binding Cassette Transporters P-Gp, BCRP
and MRP2 Among Registered Drugs. Pharm. Res..

[47] Sjöstedt N., Holvikari K., Tammela P., Kidron H. (2017). Inhibition of Breast
Cancer Resistance Protein and Multidrug Resistance Associated Protein
2 by Natural Compounds and Their Derivatives. Mol. Pharmaceutics.

[48] Moinul M., Amin S. A., Jha T., Gayen S. (2022). Updated Chemical Scaffolds
of ABCG2 Inhibitors and Their Structure-Inhibition Relationships for
Future Development. Eur. J. Med. Chem..

[49] Liang L., He Y., Li Y., Yang J., Xu F., Li L., Huang J., Wang K., Zheng Q. (2020). Relationship
between
Antofloxacin Concentration and QT Prolongation and Estimation of the
Possible False-Positive Rate. Biomed. Pharmacother..

[50] Chao A., Al-Ghoul H., McEachran A. D., Balabin I., Transue T., Cathey T., Grossman J. N., Singh R. R., Ulrich E. M., Williams A. J., Sobus J. R. (2020). In Silico
MS/MS Spectra for Identifying
Unknowns: A Critical Examination Using CFM-ID Algorithms and ENTACT
Mixture Samples. Anal. Bioanal. Chem..

[51] Gupta A., Dai Y., Vethanayagam R. R., Hebert M. F., Thummel K. E., Unadkat J. D., Ross D. D., Mao Q. (2006). Cyclosporin A, Tacrolimus
and Sirolimus Are Potent Inhibitors of the Human Breast Cancer Resistance
Protein (ABCG2) and Reverse Resistance to Mitoxantrone and Topotecan. Cancer Chemother. Pharmacol..

[52] Heo Y.-A. (2021). Voclosporin:
First Approval. Drugs.

[53] Trivedi A., Sohn W., Kulkarni P., Jafarinasabian P., Zhang H., Spring M., Flach S., Abbasi S., Wahlstrom J., Lee E., Dutta S. (2021). Evaluation
of Drug-Drug
Interaction Potential between Omecamtiv Mecarbil and Rosuvastatin,
a BCRP Substrate, with a Clinical Study in Healthy Subjects and Using
a Physiologically-Based Pharmacokinetic Model. Clin. Transl. Sci..

[54] Balasubramanian R., Maideen N. M. P. (2021). HMG-CoA Reductase Inhibitors (Statins) and Their Drug
Interactions Involving CYP Enzymes, P-Glycoprotein and OATP Transporters-an
Overview. Curr. Drug Metab..

[55] Poller B., Iusuf D., Sparidans R. W., Wagenaar E., Beijnen J. H., Schinkel A. H. (2011). Differential Impact
of P-Glycoprotein (ABCB1) and Breast
Cancer Resistance Protein (ABCG2) on Axitinib Brain Accumulation and
Oral Plasma Pharmacokinetics. Drug Metab. Dispos..

