# Supplementary material for: In Silico Regression Modeling and Improved Interpretability To Predict the Transport Inhibitory Activity of Breast Cancer Resistance Protein
Source: ACS Omega. 2026 Apr 21;11(17):25254–65. doi: 10.1021/acsomega.5c12191 (PMC13150592; doi:10.1021/acsomega.5c12191)
Supplement: Supplementary file 1 [file ao5c12191_si_001.pdf]

# Supporting Information

# *In Silico* Regression Modelling and Improved Interpretability to Predict the Transport Inhibitory Activity of Breast Cancer Resistance Protein

*Kaoru Takadera,*[1,2,3,⊥] *Donny Ramadhan,*[1,4,5,*,⊥] *Reiko Watanabe,*[1,3,*] *Kenji Mizuguchi*[1,2,3,4,*]

[1]Laboratory for Computational Biology, Institute for Protein Research, The University of Osaka, Suita, Osaka 565-0871, Japan

[2]Graduate School of Pharmaceutical Sciences, The University of Osaka, Suita, Osaka 565-0871, Japan

[3]Artificial Intelligence Center for Health and Biomedical Research, National Institutes of Biomedical Innovation, Health and Nutrition, Settsu, Osaka 566-0002, Japan

[4]Graduate School of Science, The University of Osaka, Toyonaka, Osaka 560-0043, Japan

[5]Research Center for Pharmaceutical Ingredients and Traditional Medicine, National Research and Innovation Agency (BRIN), Bogor, West Java 16911, Indonesia

# Table of Contents


**Table S1.** List of Descriptors Used in the Study....................................................S3

**Table S2.** Optuna Settings for the Comprehensive Model ....................................................S4

**Table S3.** Optuna Settings for the Simplified Descriptor Model............................................S5

**Table S4.** Top 20 Important Features in the Simplified Descriptor Model (RF).....................S6

**Table S5.** Comparison of Converted Classification Metrics with Previous Studies ................S7

**Scheme S1.** Details of the Manual Curation Process for the BCRP Dataset ..............................S7

**Figure S1.** Overview of the machine learning process. ........................................................S12

**Figure S2.** Determination of the cutoff based on the Tanimoto coefficient using the 10 test sets of the simplified descriptor model.........................................................................S13

**Figure S3.** Observed $R^2$, RMSE, and MSE during feature reduction by RFE. .......................S16

**Figure S4.** Distribution of $pIC_{50}$ values in groups with or without important fingerprints......S17

**Figure S5.** Comparison of selected molecular descriptors between approved drugs with high and low structural similarity to the BCRP dataset. .....................................................S19

**Table S1.** List of Descriptors Used in the Study

| Software | Name | Dimension | Class |
|---|---|---|---|
| RDKit | RDKit Descriptors | 208 | Physicochemical Descriptors |
| Mordred | Mordred Descriptors | 1,826 | Physicochemical Descriptors |
| jCompoundMapper | AP2D<br>CATS2D<br>ECFP<br>ECFPVariant<br>MACCS<br>RAD2D<br>DFS | 4,096 | Molecular Fingerprints |
| PaDEL-Descriptor | MACCS Fingerprints<br>PubChem Fingerprints<br>Klekota–Roth Fingerprints | 166<br>880<br>4,860 | Molecular Fingerprints |

**Table S2.** Optuna Settings for the Comprehensive Model

| Algorithm | Hyperparameter | Scale | Min | Max | Categorical Candidates (or Remarks) |
|---|---|---|---|---|---|
| RF | max_depth | int | 10 | 100 | – |
| | n_estimators | int | 100 | 600 | – |
| | max_features | log | 1.00E-03 | 1 | – |
| LGBM | num_leaves | int | 16 | 256 | – |
| | max_depth | int | 3 | 10 | – |
| | min_data_in_leaf | int | 3 | 50 | – |
| | learning_rate | log | 1.00E-03 | 1.00E-01 | – |
| SVM | C | log | 1.00E-03 | 1.00E+07 | – |
| | epsilon | log | 1.00E-07 | 1.00E-04 | – |
| | gamma | log | 1.00E-11 | 1.00E+03 | – |
| ANN | hl1 | int | 10 | 500 | – |
| | hl2 | int | 5 | 50 | – |
| | hidden_layer_sizes | – | – | – | (hl1, hl2) |
| | activation | categorical | – | – | 'identity', 'logistic', 'tanh', 'relu' |
| | max_iter | – | – | – | Deterministic value: 2,000 |
| | learning_rate_init | – | – | – | Deterministic value: 0.02 |
| | alpha | – | – | – | Deterministic value: 0.0001 |
| kNN | n_neighbors | int | 1 | 15 | – |

| | | | | | |
|---|---|---|---|---|---|
| | algorithm | categorical | – | – | ‘auto’, ‘ball_tree’,’kd_tree’, ‘brute’ |
| | p | int | 1 | 2 | – |
| | leaf_size | int | 1 | 100 | – |
| RL | regression_method | categorical | – | – | 'ridge', 'lasso' |
| | ridge_alpha | float | 0.0 | 2.0 | – |
| | lasso_alpha | float | 0.0 | 2.0 | – |

**Table S3.** Optuna Settings for the Simplified Descriptor Model

| Algorithm | Hyperparameter | Scale | Min | Max |
|---|---|---|---|---|
| RF | max_depth | int | 3 | 100 |
| | n_estimators | int | 100 | 1000 |
| | max_features | log | 1.00E-02 | 1 |
| LGBM | num_leaves | int | 16 | 256 |
| | max_depth | int | 3 | 10 |
| | min_data_in_leaf | int | 3 | 50 |
| | learning_rate | log | 1.00E-03 | 1.00E-01 |
| RL | alpha | log | 1.00E-04 | 1 |
| | l1_ratio | float | 0 | 1 |

**Table S4.** Top 20 Important Features in the Simplified Descriptor Model (RF)

| Rank | Important Features | Importance |
|---|---|---|
| 1 | PubchemFP359 | 0.04953 |
| 2 | PubchemFP576 | 0.04193 |
| 3 | PubchemFP821 | 0.03215 |
| 4 | MACCSFP151 | 0.02885 |
| 5 | PubchemFP577 | 0.02476 |
| 6 | KRFP1733 | 0.02397 |
| 7 | PubchemFP16 | 0.02139 |
| 8 | KRFP3224 | 0.01984 |
| 9 | MACCSFP120 | 0.01861 |
| 10 | MACCSFP94 | 0.01681 |
| 11 | MACCSFP62 | 0.01603 |
| 12 | MACCSFP80 | 0.01543 |
| 13 | KRFP3455 | 0.01252 |
| 14 | MACCSFP119 | 0.01228 |
| 15 | KRFP3750 | 0.01183 |
| 16 | MACCSFP129 | 0.01154 |
| 17 | PubchemFP728 | 0.01063 |
| 18 | PubchemFP196 | 0.00979 |
| 19 | PubchemFP365 | 0.00971 |
| 20 | KRFP1645 | 0.00961 |

The top five features were consistent with those identified by SHAP analysis, and many of the same features were also ranked within the top 20.

**Table S5.** Comparison of Converted Classification Metrics with Previous Studies

| Model | MCC | Balanced Accuracy |
|---|---|---|
| Jiang et al. (2020) | 0.812 | 0.905 |
| Huang et al. (2022) | 0.63 | 0.82 |
| The Best Simplified Descriptor Model | 0.68 | 0.75 |

**Scheme S1.** Details of the Manual Curation Process for the BCRP Dataset

The curation process was conducted by combining automated data processing with Python, data analysis using Excel, and manual examination of the original publications.

**Initially Collected Data**

A total of 1,572 $IC_{50}$ data points and 2,799 BCRP inhibition data points were extracted from the ChEMBL database and Jiang et al., respectively. For the ChEMBL-derived data, 1,572 $IC_{50}$ values with experimental information were retrieved using the keyword CHEMBL5393 (Type: SINGLE PROTEIN; Preferred Name: ATP-binding cassette sub-family G member 2). These data included molecular structures described in SMILES format, reported $IC_{50}$ values along with "standard relation" and "unit," and experimental details provided in the columns "assay description," "assay type," "assay organism," "assay tissue," "assay cell type," "ChEMBL ID," and "data validity comment." For the data from Jiang et al., 2,799 BCRP inhibition data entries with structural information in SMILES format were obtained. The dataset by Jiang et al. had already been

carefully curated, and a single value for each compound was selected, accompanied by a citation to the original publication.

## 1. Data Curation for ChEMBL-derived Data

a) Tagging of Experimental Information

Information on experimental conditions was extracted from the “assay description” and “assay tissue” columns of the ChEMBL-derived data. The type of experiment, substrate, and cell used for each entry were then tagged. The types of tags are as follows:

| Information Type | Tag Name |
|---|---|
| Experiment Type 1 | ‘membrane-based’*, ‘cell-based’, |
| Experiment Type 2 | ‘MTT’, ‘GFP-tagged’, ‘multi-drug’ |
| Substrate | ‘hoechst’, ‘mitoxantrone’, ‘topotecan’, ‘pheophorbide’, ‘mtx’, ‘pha’, ‘quercetin’, ‘3h-estron-3-sulfate’, ‘imatinib’ |
| Cell | ‘mdck2’, ‘mcf7’, ‘hek’, ‘ncl-h460’, ‘mcf-7’, ‘mdck’, ‘h460-mx20’, ‘s1-m1-80’, ‘k562’, ‘sf9’, ‘t8’, ‘caco’, ‘high5’, ‘h460/mx20’, ‘plb-985’, ‘kbv’, ‘saos’, ‘mcf-7/topo’, ‘mcf-mx’ |

* This tag was assigned to data with the cell type “Sf9” or “High5” (typically used for vesicular transport assays) when the assay description contained the words “membrane” or “vesicle”.

b) Filtering Data from Unexpected Experimental Conditions

Filtering was conducted based on the following criteria:

| Treatment | Filtering Parameter | Applied Condition | Target Value(s) | Reason for Exclusion |
|---|---|---|---|---|
| Remove | SMILES, Units | Blank | – | Reason 1 |
| | Standard Relation | Not equal to “=” | – | Reason 2 |
| | Data Validity Comment | “Outside typical range” | – | Reason 3 |
| | Experiment Type 1 | Contains | “membrane-based” | Reason 4 |
| | Experiment Type 2 | Contains | “MTT”, “GFP-tagged”, “multi-drug” | Reason 5 |
| | Cell | Contains | “mcf-7/topo”, “mcf-mx”, “h460/mx20” | Reason 6 |

Reason 1. Data without structural information or units cannot be used.

Reason 2. To restrict the dataset to entries with numerical activity values.

Reason 3. To exclude suspicious data flagged by ChEMBL as being outside the typical range for the corresponding activity type.

Reason 4. To exclude data obtained from membrane-based assays.

Reason 5. To exclude data derived from studies involving multiple drugs or using different evaluation criteria, such as cell viability or GFP fluorescence intensity.

Reason 6. To exclude data obtained from cell lines with altered properties due to drug resistance.

c) Standardization of $IC_{50}$ Units to μM

d) Handling of multiple $IC_{50}$ values for a single compound was performed by calculating the average $IC_{50}$ value and confirming consistency within a three-fold error range through manual inspection of the original sources.

When multiple records were available for a single compound, the average $IC_{50}$ value and the fold difference between the maximum and minimum values were calculated. In total, 210 compounds had multiple $IC_{50}$ records in the ChEMBL-derived dataset, and 203 of these (96.7%) fell within a three-fold range. The original sources of the seven compounds showing more than a three-fold difference were manually checked and corrected when errors were identified. Data series in which the inhibitor or substrate concentration was intentionally varied, or in which endpoint and real-time measurements were mixed, were removed. Data with large errors were also excluded when the reason for the discrepancy could not be determined. After intensive manual curation, the averaged $IC_{50}$ value was used for compounds with multiple records. In total, $IC_{50}$ values for 670 compounds were obtained from the ChEMBL-derived dataset. These data were generated using four types of substrates (Hoechst, Pheophoribide, Mitoxantrone, and 3h-estron-3-sulfate) and nine cell lines (MDCK, MDCKII, MCF-7, HEK, h460, Caco-2, K562, PLB-985, and SAOS).

## 2. Data Curation for Jiang et al.-derived Data

The dataset from Jiang et al. had already been carefully curated. For compounds with multiple records, the $IC_{50}$/$EC_{50}$ value was selected or averaged, with a citation to the original publication. Initially, 613 $IC_{50}$ and $EC_{50}$ values were filtered. The $EC_{50}$ values were treated as $IC_{50}$, representing the compound concentration that produces half-maximal inhibition, after confirming that these $EC_{50}$ values reflected inhibitor potency as described in the original paper. Records with blank values were removed, and only numerical data were retained. The units were standardized to μM.

As a result, $IC_{50}$ values for 565 compounds (including 57 originally reported as $EC_{50}$) were obtained from Jiang et al.

## 3. Integrating Data from ChEMBL and Jiang et al. and Verifying Consistency within a Three-fold Range through Manual Confirmation

SMILES in the ChEMBL (670 compounds) and Jiang et al. (565 compounds) datasets were standardized using MELLODY-TUNER. The two datasets were then merged by matching the standardized SMILES. A total of 363 compounds overlapped between the two datasets, and the average and fold-change between their $IC_{50}$ values were calculated. Among them, 358 compounds (98.1%) were within a three-fold range. The $IC_{50}$ values of five compounds that exceeded this range were found to be based on multiple sources in Jiang et al. The experimental conditions of these compounds were manually checked, and the $IC_{50}$ values in Jiang et al. were recalculated after excluding data obtained under unexpected conditions (e.g., MTT assays). After this curation step, 2 compounds that still showed discrepancies greater than three-fold were excluded; consequently,

all remaining data were within a three-fold range, and the averaged values were used in the final dataset. The final curated dataset comprised 870 compounds.

## 4. Conversion of Finalized $IC_{50}$ Values to $pIC_{50}$ Values

The final dataset contained 870 distinct compounds with structural information and corresponding $IC_{50}$ values. Hereafter, this dataset is referred to as the "BCRP dataset."

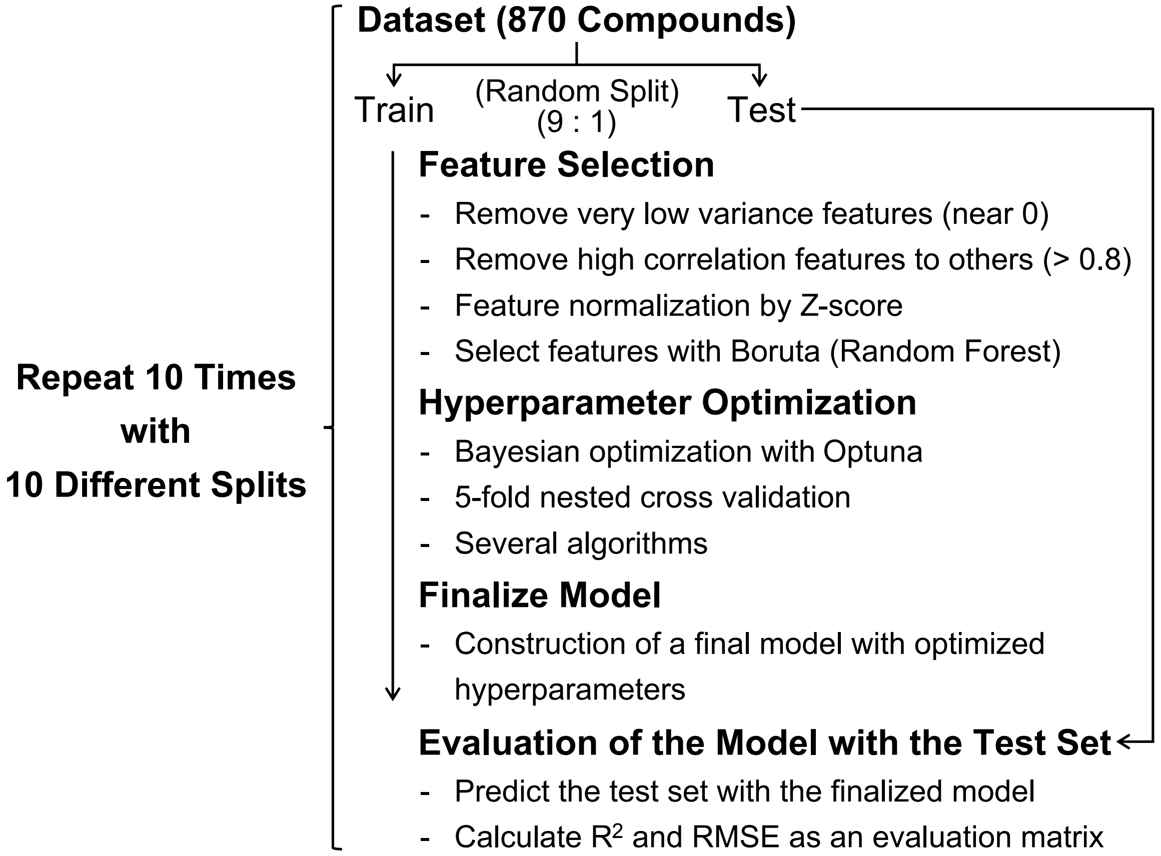


**Figure S1.** Overview of the machine learning process.

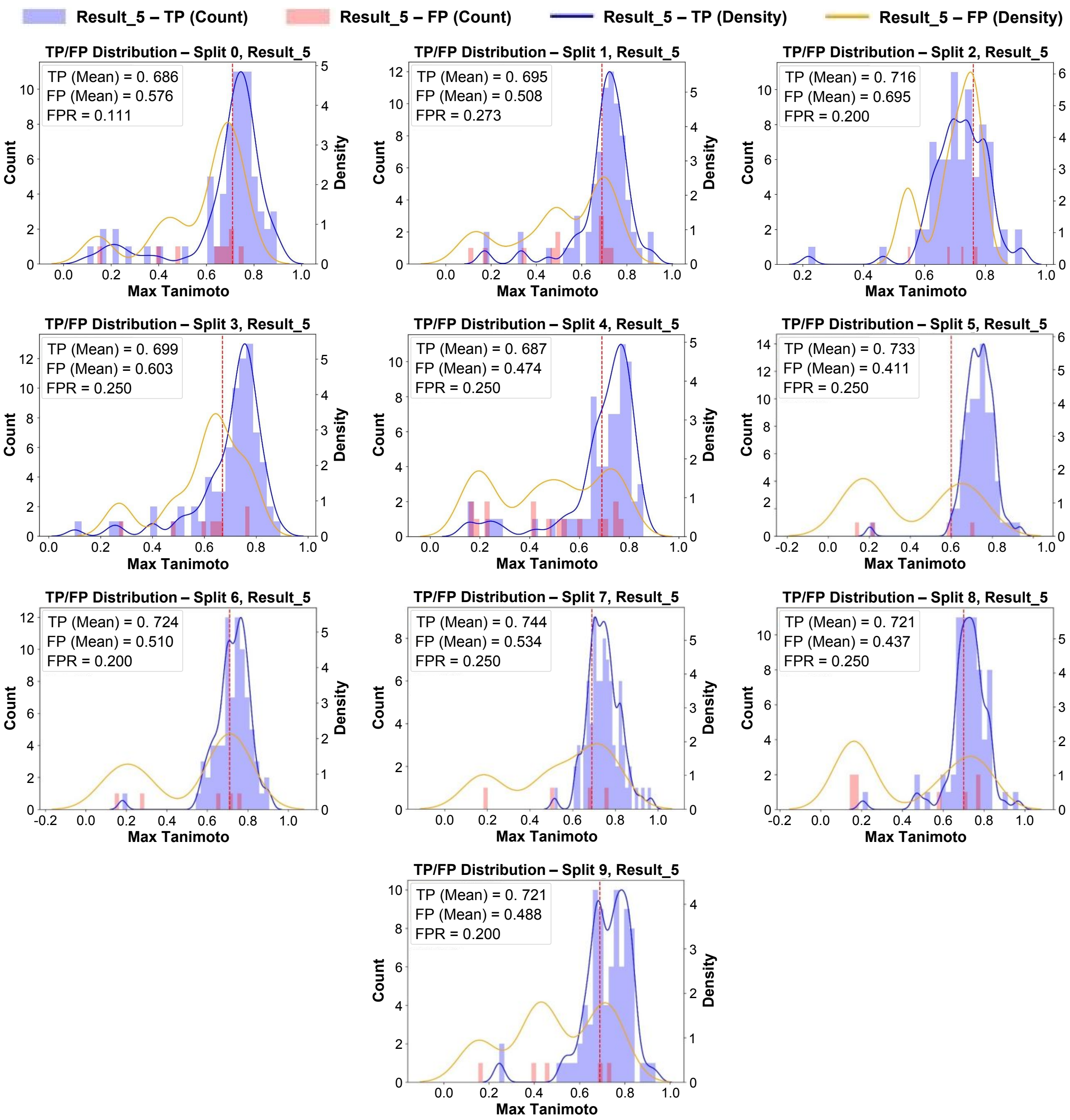


**Figure S2.** Determination of the cutoff based on the Tanimoto coefficient using the 10 test sets of the simplified descriptor model.

The maximum Tanimoto similarity between each compound in the test sets and those in the training sets was calculated. A histogram and density plot of true positive (TP) and false positive

(FP) groups across the 10 models were generated. A threshold of $pIC_{50} = 5$ was used to distinguish inhibitors from non-inhibitors. TP, true positive; FP, false positive; wide blue bar, number of compounds classified as TP; wide orange bar, number of compounds classified as FP; blue and orange lines, density of compounds in the TP and FP groups, respectively; TP_means and FP_means, mean maximum Tanimoto similarity values of the TP and FP groups; FPR, false positive rate.

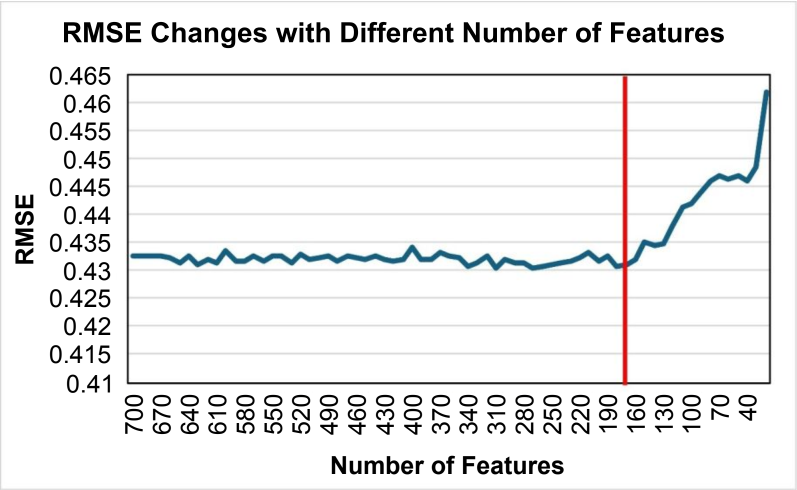
RMSE Changes with Different Number of Features
RMSE
0.465
0.46
0.455
0.45
0.445
0.44
0.435
0.43
0.425
0.42
0.415
0.41
700 670 640 610 580 550 520 490 460 430 400 370 340 310 280 250 220 190 160 130 100 70 40
Number of Features

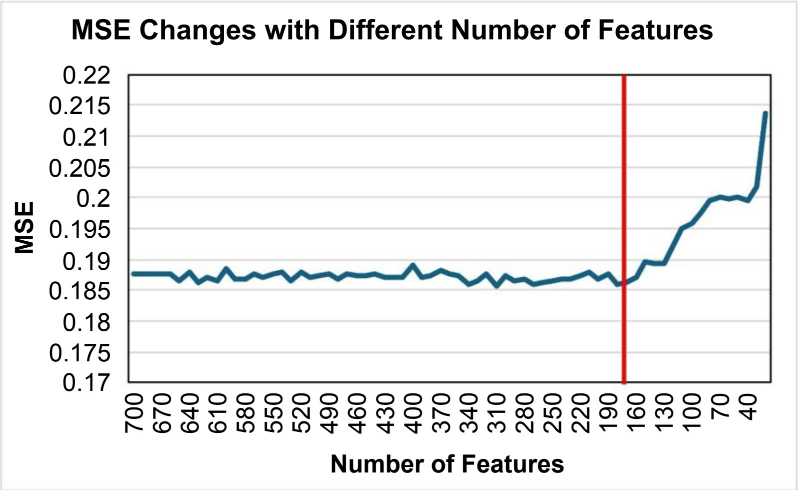
MSE Changes with Different Number of Features
MSE
0.22
0.215
0.21
0.205
0.2
0.195
0.19
0.185
0.18
0.175
0.17
700 670 640 610 580 550 520 490 460 430 400 370 340 310 280 250 220 190 160 130 100 70 40
Number of Features

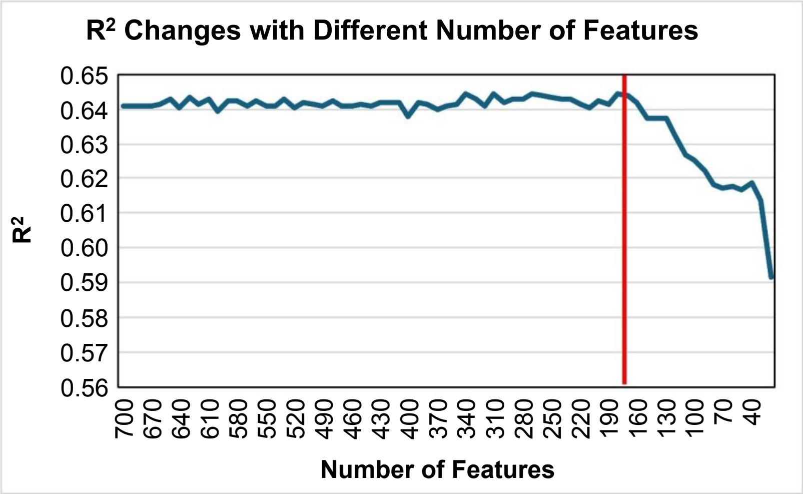
R2 Changes with Different Number of Features
R2
0.65
0.64
0.63
0.62
0.61
0.60
0.59
0.58
0.57
0.56
700 670 640 610 580 550 520 490 460 430 400 370 340 310 280 250 220 190 160 130 100 70 40
Number of Features

**Figure S3.** Observed $R^2$, RMSE, and MSE during feature reduction by RFE. MSE: mean squared error; RMSE: root mean squared error.

The number of features (n = 170) was selected based on knee/elbow point detection using the kneed package and was adopted as the final feature set in the simplified descriptor model. Red line: number of features = 170.

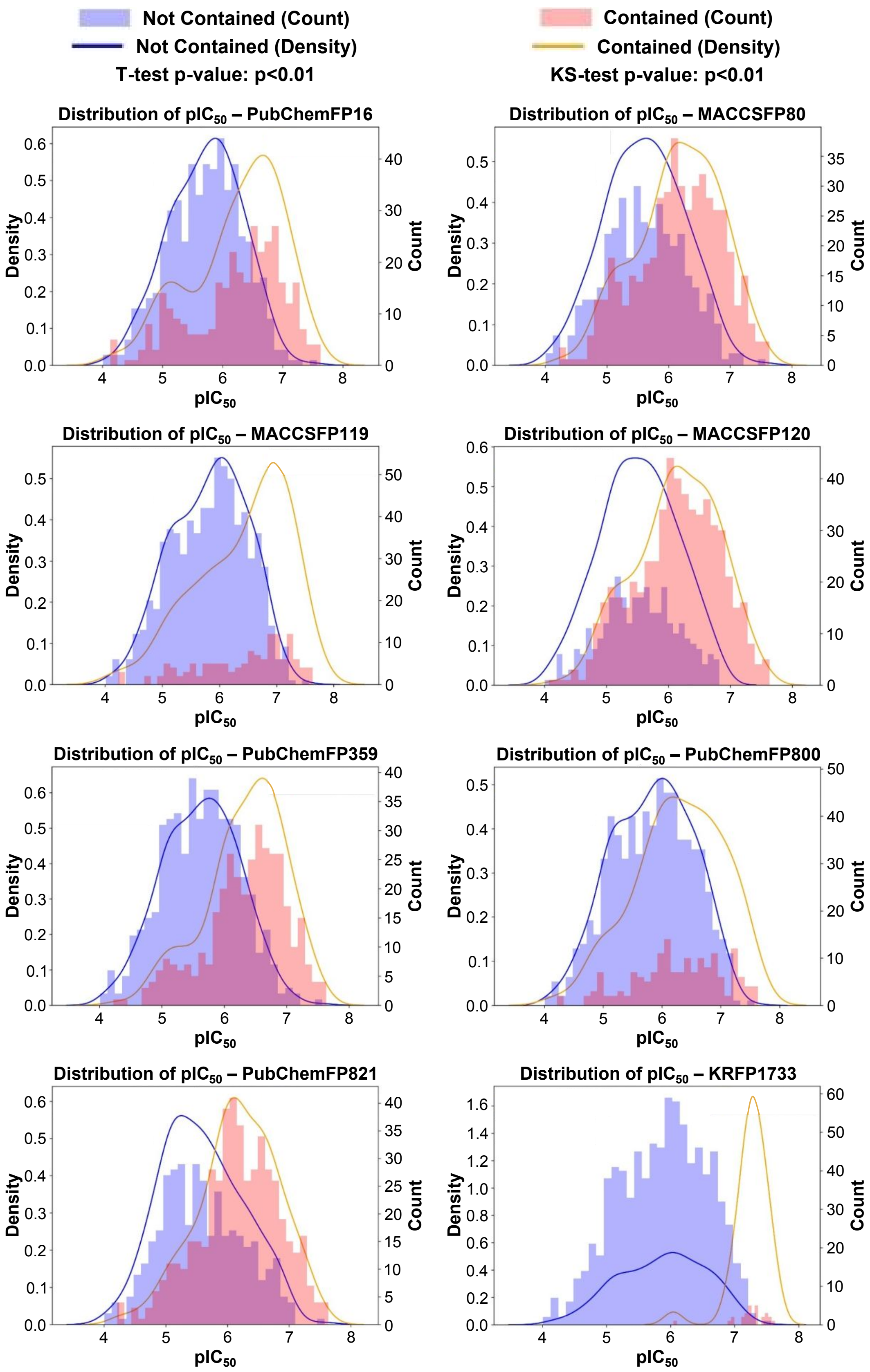


**Figure S4.** Distribution of $pIC_{50}$ values in groups with or without important fingerprints.

Bars represent the number of compounds in the group containing (red) or not containing (blue) each fingerprint. Lines indicate the compound density in the group containing (red) or not containing (blue) the fingerprint. For all panels, both the Student's t-test and Kolmogorov–Smirnov (K–S) test yielded p-values $< 0.01$.

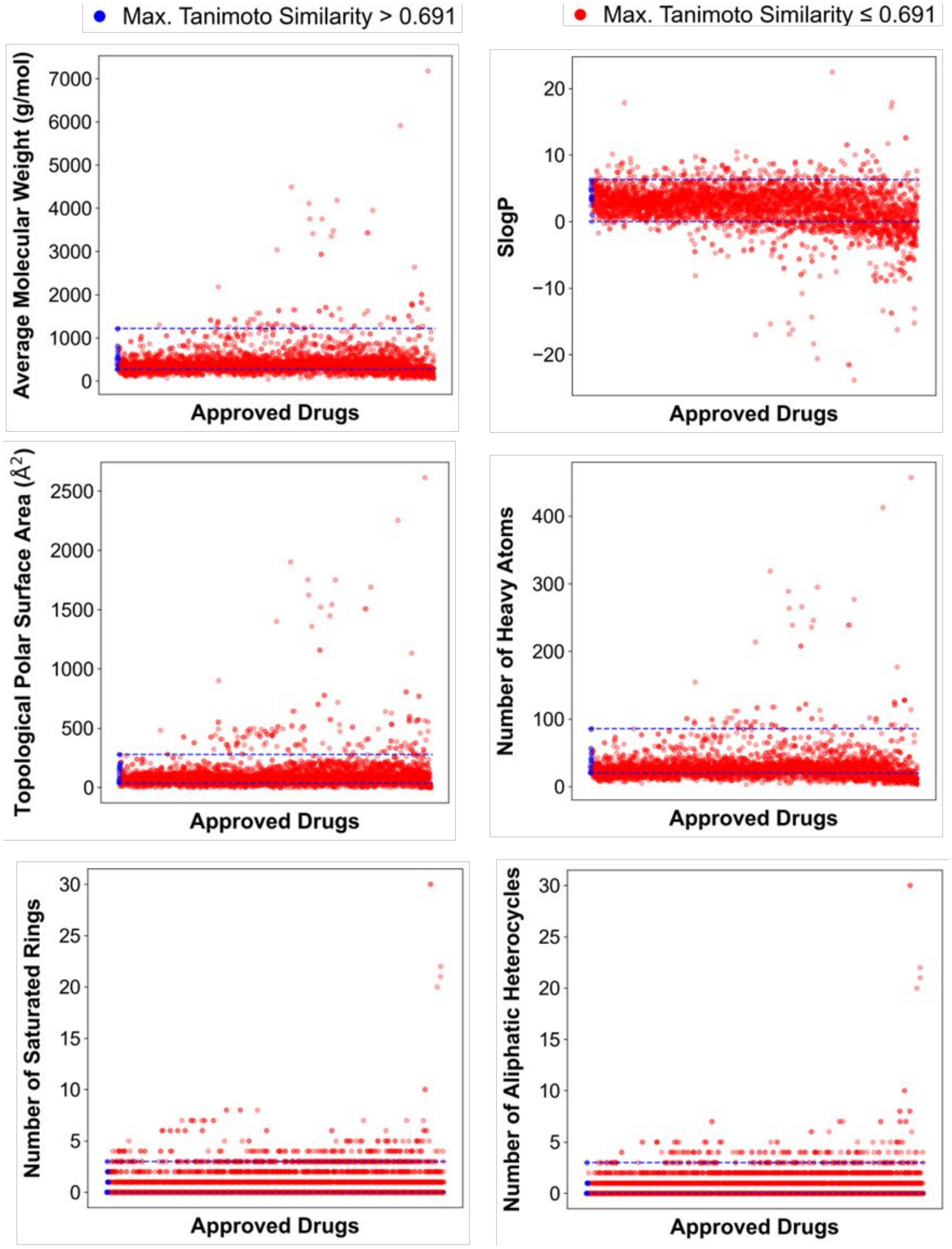


**Figure S5.** Comparison of selected molecular descriptors between approved drugs with high and low structural similarity to the BCRP dataset.

Regions within the dashed lines indicate the chemical features of compounds inside the similarity-based applicability domain.
